# Molecular basis for t^6^A modification in human mitochondria

**DOI:** 10.1093/nar/gkaa093

**Published:** 2020-02-12

**Authors:** Jing-Bo Zhou, Yong Wang, Qi-Yu Zeng, Shi-Xin Meng, En-Duo Wang, Xiao-Long Zhou

**Affiliations:** 1 State Key Laboratory of Molecular Biology, CAS Center for Excellence in Molecular Cell Science, Shanghai Institute of Biochemistry and Cell Biology, Chinese Academy of Sciences, University of Chinese Academy of Sciences, 320 Yue Yang Road, Shanghai 200031, China; 2 School of Life Science and Technology, ShanghaiTech University, 100 Hai Ke Road, Shanghai 201210, China; 3 Biology Department, College of Science, Purdue University, 150 N. University St, West Lafayette, IN 47907, USA

## Abstract

*N* ^6^-Threonylcarbamoyladenosine (t^6^A) is a universal tRNA modification essential for translational accuracy and fidelity. In human mitochondria, YrdC synthesises an l-threonylcarbamoyl adenylate (TC-AMP) intermediate, and OSGEPL1 transfers the TC-moiety to five tRNAs, including human mitochondrial tRNA^Thr^ (hmtRNA^Thr^). Mutation of hmtRNAs, YrdC and OSGEPL1, affecting efficient t^6^A modification, has been implicated in various human diseases. However, little is known about the tRNA recognition mechanism in t^6^A formation in human mitochondria. Herein, we showed that OSGEPL1 is a monomer and is unique in utilising C34 as an anti-determinant by studying the contributions of individual bases in the anticodon loop of hmtRNA^Thr^ to t^6^A modification. OSGEPL1 activity was greatly enhanced by introducing G38A in hmtRNA^Ile^ or the A28:U42 base pair in a chimeric tRNA containing the anticodon stem of hmtRNA^Ser^(AGY), suggesting that sequences of specific hmtRNAs are fine-tuned for different modification levels. Moreover, using purified OSGEPL1, we identified multiple acetylation sites, and OSGEPL1 activity was readily affected by acetylation via multiple mechanisms *in vitro* and *in vivo*. Collectively, we systematically elucidated the nucleotide requirement in the anticodon loop of hmtRNAs, and revealed mechanisms involving tRNA sequence optimisation and post-translational protein modification that determine t^6^A modification levels.

## INTRODUCTION

Mitochondria are the powerhouses of most eukaryotic cells, except some lower eukaryotes, such as *Giardia lamblia* (1). One of the main functions of mitochondria is to provide ATP via oxidative phosphorylation, carried out by proteins of five respiratory chain complexes, of which four contain a unique combination of both nuclear and mitochondrial DNA (mtDNA)-encoded protein subunits (2,3). Therefore, mitochondria finely integrate nuclear and mitochondrial genetic systems, and hemostasis is pivotal for various cellular activities, without which, mitochondrial dysfunctions can disrupt ATP generation and lead to pathologies (2,3). Human mtDNA harbors 37 genes encoding 22 mitochondrial tRNAs, 2 rRNAs (12S and 16S) and 13 mRNAs (4). These 13 mtDNA-derived proteins are synthesized within the organelle using the mitochondrial translation apparatus, and all are components of the respiratory chain complexes.

Mitochondrial tRNAs must undergo maturation before genetic decoding, and one of the key maturation processes is tRNA modification. In bovine mitochondrial tRNAs, 15 types of modifications have been identified at 118 positions (5). These modifications mainly occur at the anticodon loop region, especially at bases 34 and 37, and play important roles in stabilising the tRNA tertiary structure, fine-tuning the decoding properties of tRNA, and coordinating the binding of tRNA to protein factors during translation (6).

*N* ^6^-Threonylcarbamoyladenosine (t^6^A) is a highly conserved modification present in nearly all ANN-decoding (N = A, T, G, C) tRNAs, and is one of the few modifications found in all three domains of life (7–9). Comparative genomic analyses have revealed the involvement of TsaC/Sua5/YrdC and TsaD/Kae1/Qri7 protein families in the t^6^A biosynthesis pathway (10–13). However, the detailed biogenesis of t^6^A remains elusive. The proposed modification pathway generally involves two steps; formation of an intermediate l-threonylcarbamoyl adenylate (TC-AMP), and subsequent transfer of the TC-moiety of TC-AMP onto A37 of tRNA substrates. In bacteria, the TsaC (the bacterial Sua5/YrdC orthologue) enzyme first synthesises the TC-AMP intermediate, and together with TsaB and TsaE (two bacteria specific proteins), TsaD (the bacterial Kae1/Qri7 orthologue) then transfers the TC-moiety from TC-AMP onto tRNA (13–15). TsaD must bind to TsaB for efficient catalysis, while an additional TsaE subunit seems to facilitate multiple rounds of modification via hydrolysis of ATP (14,15). In archaea and eukaryotic cytoplasm, Sua5/YrdC (a monomeric protein) produces TC-AMP, and transfer is performed by the Kae1/OSGEP subunit of the KEOPS (Kinase, putative Endopeptidase and Other Proteins of Small size) complex, which is also involved in other processes including telomere replication and recombination, transcription, and chromosome segregation (16–22). The archaeal KEOPS complex is a duplicated linear arrangement of four proteins (Pcc1-Kae1-Bud32-Cgi121) (23), whereas the yeast/human KEOPS complex is a linear complex of Gon7/C14orf142-Pcc1/LAGE3-Kae1/OSGEP-Bud32/TP53RK-Cgi121/TPRKB (19,20,24). In yeast mitochondria, mitochondria-localised Sua5 and Qri7 jointly generate the t^6^A modification of mitochondrial tRNAs, and Qri7 must be dimeric for t^6^A modification (25). It is notable that in all studied cases, the catalytic subunit (bacterial TsaD, yeast cytoplasmic Kae1, and mitochondrial Qri7) has to form a heterodimer (TsaD/TsaB; Kae1/Pcc1) or homodimer (Qri7/Qri7) for modification to take place (14,15,23,25–28). Recently, YrdC (yeast Sua5 homolog) and OSGEPL1 (*O*-sialoglycoprotein endopeptidase-like protein 1) (yeast Kae1/Qri7 homolog) were identified as human mitochondrial t^6^A-modification enzymes (29).

Previous studies established that the t^6^A modification facilitates correct anticodon–codon pairing by promoting base stacking and preventing intraloop base pairing of U33:A37, resulting in enhanced translation fidelity (30,31). Other studies also suggest critical roles in promoting aminoacylation efficiency (29), preventing frameshifting during decoding (32) and facilitating modification at other sites (33). Consistently, deletion of catalytic Kae1 in yeast leads to defective t^6^A modification and impaired cell growth (10). Even knockout of non-t^6^A catalytic subunits in the KEOPS complex, such as Pcc1 and Bud32, abolishes t^6^A generation and disrupts cell growth. Interestingly, a cytoplasmic localised Qri7 lacking a mitochondrial targeting sequence (MTS) was found to complement defective t^6^A modification due to knockout of Kae1 or TsaD genes *in vivo* (10), suggesting a universal t^6^A modification mechanism in yeast cytoplasm and mitochondria, and indicating that yeast is a suitable model for studying the catalytic mechanism of mitochondrial Qri7. Like Sua5 in yeast, YrdC in human cells is localized in both the cytoplasm and mitochondria, and deletion is fatal, possibly due to simultaneous loss of cytoplasmic and mitochondrial t^6^A formation (29). By contrast, Qri7/OSGEPL1 is solely localized in mitochondria (34). *OSGEPL1* knockout cells exhibit respiratory defects and reduced mitochondrial translation (29), suggesting a critical role for mitochondrial t^6^A modification in mitochondrial metabolism. Indeed, genetic mutations in t^6^A modification-related genes have been linked to various human diseases. For example, mutations in *OSGEP*, *TP53RK*, *TPRKB*, *LAGE3* and *C14orf142*, encoding all five subunits of the human cytoplasmic KEOPS complex, lead to defective t^6^A modification and Galloway-Mowat syndrome (GAMOS), characterised by early-onset nephrotic syndrome, microcephaly and brain anomalies (35). Additionally, a separate study showed that the *OSGEP* gene c.974G>A mutation is associated with neurodegeneration and renal tubulopathy (36). Very recently, several *YrdC* mutations were found to also cause GAMOS with a more severe phenotype than the KEOPS mutations, probably due to simultaneous loss of cytoplasmic and mitochondrial t^6^A formation (37). Consistently, the *OSGEPL1* gene c.1143A>TA (p.Leu378PhefsTer3) mutation appears to be highly pathogenic in angioimmunoblastic T-cell lymphoma, but details of its pathogenesis remain unknown (38).

Despite identification of enzymes related to t^6^A modification in all three domains of life, details of the modification mechanisms, including tRNA recognition by Kae1/Qri7/OSGEPL1, remain limited. This is largely due to difficulties in successfully reconstituting an efficient t^6^A modification activity *in vitro* using multiple types of enzyme subunits. In one study using a *Xenopus laevis* oocytes *in vivo* modification system, besides A37, only U36 was absolutely required for efficient t^6^A modification (39). Our recent work revealed that, at least for human mitochondrial tRNA^Thr^ (hmtRNA^Thr^), A38 is a prerequisite for t^6^A modification, and it cannot be replaced by the other three bases (40). Our results were later confirmed by another group who showed that a pathogenic hmtRNA^Thr^-A38G variant was defective in t^6^A modification *in vivo* (29). However, the functions of other bases in selection and recognition, especially those in the anticodon loop region, remain unknown. Furthermore, except for the two His residues in Kae1 (10,41), the amino acid resides in Kae1/Qri7/OSGEPL1 that mediate tRNA recognition and catalysis remain to be determined.

To reveal the mechanism of the modification enzymes to recognize tRNA and potential key amino acid residues of the enzymes in t^6^A modification, we selected human YrdC, OSGEPL1 and hmtRNAs as a model system. Mitochondrial t^6^A modification utilises simple components, and only requires Sua5 and Qri7 in yeast, making it an ideal system for activity reconstitution (25). Indeed, we recently purified Sua5 and Qri7 and studied the potential pathogenic mechanism of hmtRNA^Thr^ (40). Five bovine or human mitochondrial tRNAs with t^6^A modification have been identified (5,29), along with human mitochondrial t^6^A modification enzymes YrdC and OSGEPL1 (29). Human mitochondrial t^6^A defects have been firmly linked with diseases (35,37,38). Furthermore, t^6^A is essential for both mitochondrial and non-mitochondrial tRNAs. Therefore, clarification of human mitochondrial t^6^A modification would help to understand mechanism of t^6^A modification of non-mitochondrial tRNAs and etiology of related human diseases. In the present work, using [^14^C]Thr and tRNA transcripts, we elucidated the determinants and anti-determinants within the tRNA anticodon loop of hmtRNA^Thr^ (the best substrate of YrdC and OSGEPL1, see results below) in human mitochondrial t^6^A modification and revealed that human mitochondrial t^6^A modification is affected by both the tRNA structure itself, and by post-translational acetylation of OSGEPL1.

## MATERIALS AND METHODS

### Materials

l-Thr, NTP, GMP, tetrasodium pyrophosphate, pyrophosphatase (PPiase), Tris-base, MgCl_2_, MnCl_2_, NaCl, DTT, NaHCO_3_, activated charcoal, anti-FLAG (F7425), anti-GAPDH (G8795) antibodies, horseradish peroxidase (HRP)-conjugated secondary antibodies, standard proteins (including bovine serum albumin, ovalbumin, carbonic anhydrase, ribonuclease A and aprotinin) and biotinamidohexanoic acid hydrazide (B3770-25MG) were purchased from Sigma (St. Louis, MO, USA). Anti-Myc (HOA012MC), anti-HA (HOA012HA) and anti-His_6_ (HOA012HS) were purchased from Shanghai HuiOu Biotechnology Co. Ltd (Shanghai, China). [α-^32^P]ATP, [^14^C]Thr was obtained from Perkin Elmer Inc. (Waltham, MA, USA). KOD-plus mutagenesis kits were obtained from TOYOBO (Osaka, Japan). Yeast was transformed using a Yeastmaker Yeast Transformation System 2 kit (Takara Bio, Japan). Lipofectamine 2000 transfection reagent, SuperSignal West and Dynabeads protein G were obtained from Thermo Scientific (Waltham, MA, USA). Ni^2+^-NTA Superflow resin was purchased from Qiagen Inc. (Hilden, Germany). Polyethyleneimine cellulose plates were purchased from Merck (Darmstadt, Germany). Primer synthesis and DNA sequencing were performed by Biosune (Shanghai, China).

### Plasmid construction, mutagenesis and gene expression

Genes encoding Qri7 (UniProt No. P43122), YrdC (UniProt No. Q86U90) and OSGEPL1 (UniProt No. Q9H4B0) were amplified separately from yeast genomic DNA or cDNA obtained by reverse transcription of total RNA from human embryonic kidney 293T (HEK293T) cells. *Qri7* was then inserted between BamHI and XhoI sites of pCMV-3Tag-3A and pCMV-3Tag-4A, and *YrdC* was inserted between BamHI and *Xho*I sites of pcDNA3.1 with a C-terminal HA-tag, and pET28a with an N-terminal His_6_-tag, respectively. *OSGEPL1* and its variants were inserted in the gap between BamHI and XhoI sites of pCMV-3Tag-3A, pCMV-3Tag-4A and p425TEF-C-His (with a fragment encoding a C-terminal His_6_-tag for western blot detection), respectively. Mature OSGEPL1 (Leu^35^-Ile^414^) without the MTS (Met^1^-Phe^34^) (29) and its variants were also inserted between BamHI and XhoI sites of pET28a. Yeast *Sua5* and *Qri7* genes were constructed as reported previously (40). Yeast Kae1 (UniProt No. P36132) and human OSGEP (UniProt No. Q9NPF4) were inserted between *Spe*I and *Sal*I sites in the p425TEF-C-His vector. Gene fragment encoding mature form of human mitochondrial seryl-tRNA synthetase (UniProt No. Q9NP81; hmSerRS, Thr^35^-Ser^518^), as deduced from the MTS cleavage site of bovine mitochondrial SerRS (42), was cloned between NdeI and NotI sites in the pET28a vector. Primers used for cloning are listed in the Supplementary Table S1. *YrdC* and mature *OSGEPL1* genes were expressed in *Escherichia coli* Rosetta (DE3) cells. *YrdC* overexpression was induced with 200 μM isopropyl β-d-1-thiogalactopyranoside (IPTG) when the initial cell culture reached an absorbance at 600 nm (*A*_600_) of 0.6, and transformants were cultured overnight at 18°C. Meanwhile, genes encoding OSGEPL1 and its mutants were induced with 150 μM IPTG and transformants were cultured overnight at 30°C. Both human cytoplasmic and mitochondrial lysyl-tRNA synthetases are encoded by the same gene via mRNA alternative splicing; therefore, they are nearly identical except for minor differences in the very N-terminus (43). Expression of the gene encoding human lysyl-tRNA synthetase (hLysRS) (UniProt No. Q15046) was performed as described in a previous report (44). Expression of the hmSerRS gene was induced with 50 μM IPTG overnight at 18°C. Protein purification was performed according to previously described methods (45,46), except for eluted OSGEPL1, and its mutants and hmSerRS, which were concentrated and further purified by gel filtration on a Superdex 75 or Superdex S200 column. Protein concentration was determined using a Protein Quantification Kit (BCA Assay, Beyotime, Shanghai, China) under the guidance of the manufacturer, and the molar absorption coefficient was calculated according to the sequence of each protein (47).

### tRNA gene cloning and transcription

Genes encoding hmtRNA^Thr^, hmtRNA^Ser^(AGY), *Saccharomyces cerevisiae* cytoplasmic tRNA^Thr^(AGU) (*Sc*tRNA^Thr^(AGU)), tRNA^Thr^(CGU) (*Sc*tRNA^Thr^(CGU)), *S. cerevisiae*mitochondrial tRNA^Arg^(UCU) (*Sc*mtRNA^Arg^(UCU)) were incorporated into the pTrc99b plasmid, while those encoding hmtRNA^Ile^ and hmtRNA^Asn^ were recombined with pTrc99b together with hammerhead ribozymes to improve transcription efficiency. tRNA transcripts were obtained by *in vitro* transcription as described previously (48,49). During transcription of tRNA with a hammerhead ribozyme (50), the transcription mixture was incubated at 65°C for 1 h after digestion of DNA template to facilitate self-splicing of the transcript. A mutant of hmtRNA^Lys^, hmtRNA^Lys^-Ki (U50:A64) (with the A50:U64 in wild-type (WT) hmtRNA^Lys^ replaced with U50:A64), which has been shown to substitute well for WT hmtRNA^Lys^ (51,52), was transcribed and used in this study to avoid misfolding of WT tRNA. tRNA gene mutagenesis was performed according to the protocol provided with the KOD-plus mutagenesis kit. Primers used for template preparation are listed in the Supplementary Table S1. The tRNA concentration was determined by ultraviolet absorbance at 260 nm. The extinction coefficient was calculated from the sequence of each tRNA.

### Determination of *in vitro* t^6^A modification and aminoacylation activities

The t^6^A modification reaction was performed at 37°C in a 40 μl reaction mixture containing 50 mM Tris–HCl (pH 8.0), 200 mM NaCl, 15 mM MgCl_2_, 5 mM MnCl_2_, 50 mM NaHCO_3_, 5 mM DTT, 4 mM ATP, 100 μM [^14^C]Thr, 10 μM hmtRNAs or variants and 2 μM YrdC and OSGEPL1.

Aminoacylation time-course curves were determined as follows: a reaction mixture containing 50 mM Tris–HCl (pH 7.5), 10 mM KCl, 10 mM MgCl_2_, 2 mM DTT, 2.5 mM ATP, 200 μM [^14^C]Lys and 10 μM hmtRNA^Lys^-Ki was incubated with 200 nM hLysRS; a reaction mixture containing 60 mM Tris–HCl (pH 7.5), 15 mM KCl, 60 mM MgCl_2_, 5 mM DTT, 2.5 mM ATP, 62.1 μM [^14^C]Ser and 10 μM hmtRNA^Ser^(AGY) was incubated with 200 nM hmSerRS.

Aliquots (9 μl) of the reaction solution were added to Whatman filter pads at various time intervals and quenched with cold 5% trichloroacetic acid (TCA). Pads were washed three times for 15 min each with cold 5% TCA, then three times for 10 min each with 100% ethanol. Pads were dried under a heat lamp, and the radioactivity of precipitates was quantified using a scintillation counter (Beckerman Coulter, Atlanta, USA).

### OSGEPL1 ATPase activity assay

The ATPase activity of OSGEPL1 and its mutants was determined in a 10 μl reaction mixture containing 60 mM Tris–HCl (pH 8.0), 50 mM NaCl, 50 mM MgCl_2_, 2 mM [α-^32^P]ATP and 1 μM OSGEPL1 or its variants in the absence or presence of 10 mM FeCl_3_ at 37°C. Aliquots (1.5 μl) of the reaction mixture at various time intervals were mixed with 6 μl of stop solution (0.2 M NaAc and 1% SDS). Next, 2 μl of each mixture was spotted onto a PEI-cellulose plate and separated by thin-layer chromatography (TLC) in 0.1 M NH_4_Ac and 5% acetic acid. Plates were visualized by phosphorimaging and data were analysed using Multi-Gauge Version 3.0 software (FUJIFILM, Tokyo, Japan). Quantification of [α-^32^P]AMP was achieved by densitometry in comparison with [α-^32^P]ATP samples of known concentrations.

### Biotin labeling of hmtRNA^Thr^ and biolayer interferometry

Labelling of hmtRNA^Thr^ with biotin was carried out using our established method. Briefly, 100 μg of hmtRNA^Thr^ was incubated with 25 mM KIO_4_ in 160 μl solution at room temperature in darkness for 1 h, and the reaction was terminated by adding 100 μl 50% ethylene glycol. Next, tRNA was precipitated with three volumes of ethanol at −80°C for 2 h, and after centrifugation, the pellet was dissolved in 100 μl 10 mM biotinamidohexanoic acid hydrazide. The dissolved tRNA was incubated in the dark at 37°C for 2 h, then mixed with 100 μl 0.2 M NaBH_4_ and 200 μl 1 M Tris–HCl (pH 8.0) and incubated on ice for 30 min. Finally, hmtRNA^Thr^ was precipitated with three volumes of ethanol at −20°C overnight, and after centrifugation, the pellet was dissolved in 5 mM MgCl_2_. The labelling efficiency was confirmed by electrophoretic mobility shift assay (EMSA). Biotin-labeled hmtRNA^Thr^ (Bio-tRNA^Thr^) was used to determine the binding affinity between OSGEPL1 and hmtRNA^Thr^ using an Octet RED^96^ instrument with Bio-tRNA^Thr^ immobilized on anti-affinity streptavidin sensor tips in the presence of various concentrations of OSGEPL1 or its variants. Biolayer interferometry procedures were performed at 25°C in 200 μl reaction mixtures containing 50 mM Tris–HCl (pH 8.0), 150 mM NaCl and 0.002% Tween-20, which was also used to dilute protein samples and Bio-tRNA^Thr^. The dissociation constant (*K*_d_) was obtained by fitting the processed data to a binding/dissociation curve with the 1:1 model in the Octect analysis software (Data Analysis 9.0, ForteBio, USA), with *R*^2^ >0.99 for fitting.

### Construction of strain *Sc*Δ*Kae1* and yeast complementarity assays

Firstly, the *Kae1* open reading frame (ORF) was inserted between BamHI and SalI sites of p416TEF, and the resulting p416TEF-*Kae1* was transformed into BY4741 (Supplementary Figure S1, top right) using the Yeastmaker Yeast Transformation System 2 according to the manufacture's protocol. Secondly, the DNA fragment containing the 5′-untranslated region (5′-UTR; 160 bp) and 3′-UTR (250 bp) of *Kae1* were amplified by PCR from yeast genomic DNA. Next, the 5′-UTR was inserted between SalI and XhoI sites, and the 3′-UTR was inserted between BamHI and SalI sites of pRS303 to obtain pRS303-*Kae1*-5/3-UTR (Supplementary Figure S1, top left). Thirdly, pRS303-*Kae1*-5/3-UTR was linearised by digestion with SalI and transformed into BY4741 containing p416TEF-*Kae1* (Supplementary Figure S1, bottom). The genomic Kae1 gene was deleted via homologous recombination. Transformants were screened on a SD/Ura^−^/His^−^ plate, and selected for subsequent confirmation of loss of the chromosome-encoded *Kae1* gene by PCR amplification (Supplementary Figure S1, bottom). Accordingly, the WT *Kae1* gene was instead expressed using an introduced maintenance plasmid, p416TEF-*Kae1*, which contains a URA3 gene (encoding orotine-5′-monophosphate dicarboxylase) that is lost in the presence of 5-FOA due to its conversion to toxic fluorodeoxyuridine. The resultant *Kae1* deletion strain (*Sc*Δ*Kae1*) was further confirmed by failure to grow on the SD/His^−^/5-FOA plate. For complementation, individual genes of interest were cloned into p425TEF-C-His. Complementation was performed by transforming individual constructs into the *Sc*Δ*Kae1* strain. Transformants were selected on SD/Ura^−^/Leu^−^ plates and a single clone was cultured in liquid SD/Leu^−^ medium. The culture was diluted to a concentration equivalent to 1 OD_600_, and a 10-fold dilution of the yeast culture was plated onto a SD/Leu^−^ plate in the absence or presence of 5-FOA, to induce loss of the rescue plasmid (p416TEF-*Kae1*). Growth rates of *Sc*Δ*Kae1* expressing various genes were observed and compared. Western blotting analysis was performed before 5-FOA selection due to failed complementation by some mutants.

### Cell culture, transfection and co-immunoprecipitation (Co-IP)

HEK293T cells were cultured in Dulbecco's modified Eagle's medium supplemented with 10% fetal bovine serum in a 37°C incubator with 5% CO_2_ at a confluence of 70% before transfection using Lipofectamine 2000 transfection reagent according to the manufacturer's protocol. At 24 h after transfection, cells were harvested, washed with ice-cold phosphate-buffered saline (PBS) three times, and lysed with 1 ml of ice-cold lysis buffer (50 mM Tris–HCl pH 7.5, 150 mM NaCl, 5 mM ethylenediaminetetraacetic acid, 1% Triton X-100) supplemented with a protease inhibitor cocktail for 15 min at 4°C with rotation. The supernatant was collected by centrifugation at 12 000 × g for 30 min. Whole cell lysates were incubated with anti-FLAG or anti-HA or anti-Myc antibodies with agitation overnight, and mixtures were then incubated with Dynabeads protein G for 3 h. Recovered immune complexes were washed three times with ice-cold PBS containing 0.05% Tween-20 (PBST) buffer (137 mM NaCl, 2.7 mM KCl, 10 mM Na_2_HPO_4_, 2 mM KH_2_PO_4_, 0.05% Tween-20). Proteins were eluted from beads in 2× protein loading buffer comprising 100 mM Tris–HCl pH 7.0, 4% sodium dodecyl sulphate (SDS), 0.2% bromophenol blue, 20% glycerol and 200 mM DTT.

### Western blotting

Protein samples were separated on a 10% separating gel by SDS-PAGE and transferred to a methanol-activated polyvinylidene fluoride (PVDF) membrane, which was then blocked with 5% milk in PBST for 1 h at room temperature. Immunoblotting was performed using anti-FLAG, anti-Myc or anti-HA antibodies overnight. After washing with PBST three times, the membrane was incubated with HRP-conjugated rabbit anti-mouse IgG secondary antibody at a dilution of 1:5000 in PBST for 1 h at room temperature. Detection was performed using SuperSignal West. For silver staining and mass spectrometry (MS) analysis, the protein eluted from Co-IP samples was separated by 10% SDS-PAGE and stained with a silver stain kit for MS (Thermo Scientific) according to the manufacturer's protocol.

### Gel filtration analysis of OSGEPL1

Purified OSGEPL1 was analysed by high-performance liquid chromatography (HPLC) on a Superdex 75 column using running solution (50 mM Tris–HCl pH 8.0, 150 mM NaCl) at a rate of 0.5 ml/min. Standard proteins were also loaded and eluted under the same conditions. A linear calibration curve was obtained by plotting the logarithms of the known molecular masses of standard proteins versus their elution times.

## RESULTS

### OSGEPL1 is a monomer and does not interact with YrdC

**Figure 1.**
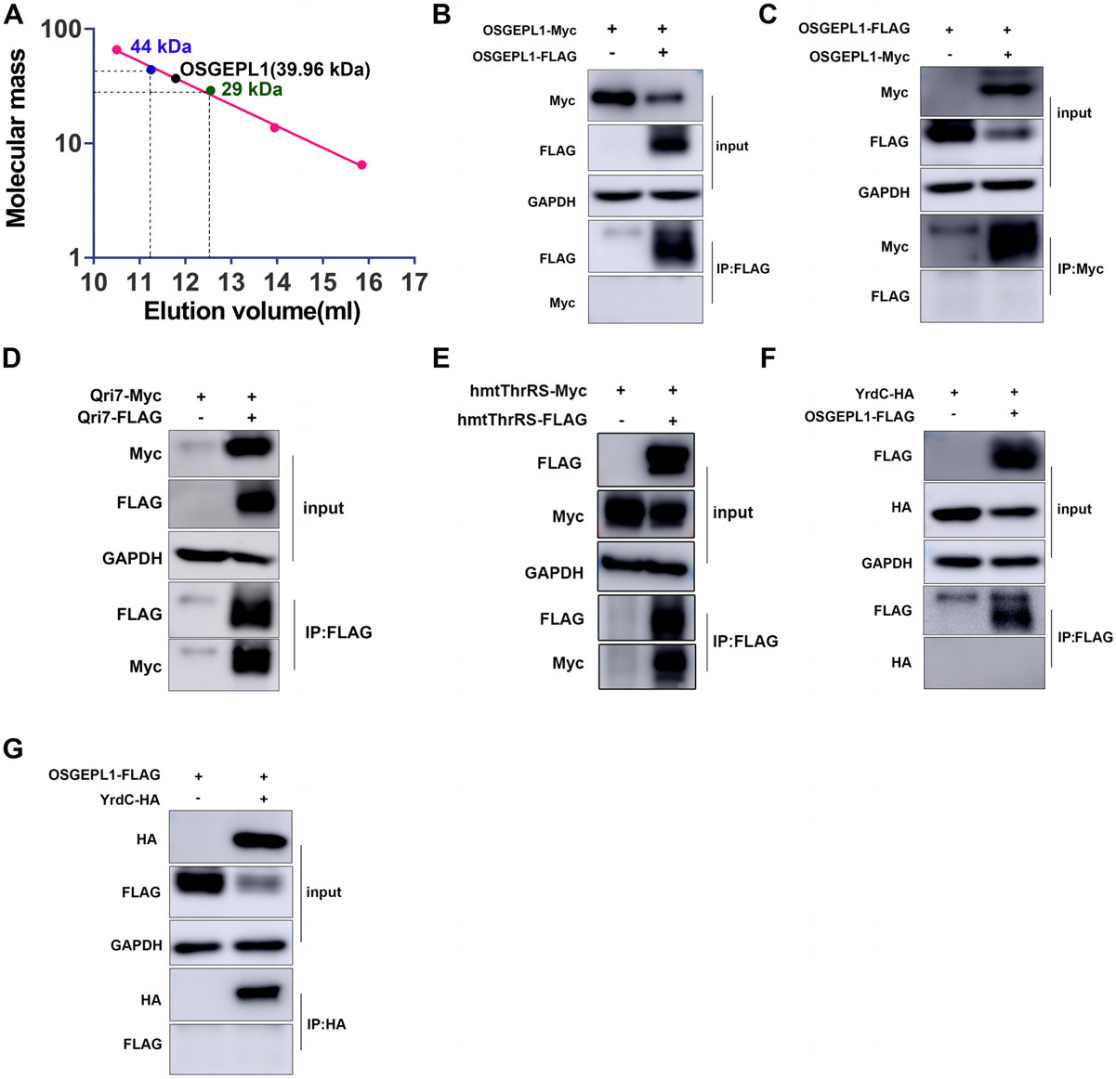
OSGEPL1 is a monomer and does not interact with YrdC. (**A**) Determination of the molecular mass of OSGEPL1 based on protein standards and elution volumes. (**B**) OSGEPL1-FLAG and OSGEPL1-Myc were co-expressed in HEK293T cells, and OSGEPL1-Myc could not be pulled down by OSGEPL1-FLAG in a Co-IP assay. (**C**) OSGEPL1-FLAG could not be precipitated by OSGEPL1-Myc in a Co-IP assay. (**D**) Dimeric Qri7 was used as a positive control. Qri7-FLAG and Qri7-Myc were co-expressed in HEK293T cells, and Qri7-Myc could be pulled down by Qri7-FLAG in a Co-IP assay. (**E**) Dimeric hmThrRS was used as a positive control, and hmThrRS-Myc was pulled down by hmThrRS-FLAG, as expected. (**F**) OSGEPL1-FLAG and YrdC-HA were co-expressed in HEK293T cells, and YrdC-HA could not be pulled down by OSGEPL1-FLAG in a Co-IP assay. (**G**) OSGEPL1-FLAG could not be precipitated by YrdC-HA in a Co-IP assay.

Full-length YrdC was purified from *E. coli* (Supplementary Figure S2A). Gel filtration analysis and co-immunoprecipitation (Co-IP; Supplementary Figure S2B, C) revealed that it was a monomer, consistent with monomeric Sua5 in archaea (53). Mature OSGEPL1 (Leu^35^-Ile^414^) without the MTS (Met^1^-Phe^34^) was purified (Supplementary Figure S2D). The calculated molecular mass of purified OSGEPL1 together with the His_6_-tag is 45.2 kDa. Its molecular mass was determined by gel filtration analysis based on the elution volumes of five standard proteins, namely bovine serum albumin (66 kDa), ovalbumin (44 kDa), carbonic anhydrase (29 kDa), ribonuclease A (13.7 kDa) and aprotinin (6.5 kDa). The determined molecular mass of OSGEPL1 was 36.96 kDa (Figure 1A). Furthermore, Co-IP was used to study the tertiary structure of OSGEPL1. Dimeric yeast Qri7 (25) and human mitochondrial ThrRS (hmThrRS), a dimeric tRNA synthetase (48), were included in Co-IP as a dimer control. Genes encoding a C-terminal FLAG-tagged OSGEPL1 (OSGEPL1-FLAG) and a C-terminal Myc-tagged OSGEPL1 (OSGEPL1-Myc) were co-expressed in HEK293T cells. Using anti-FLAG antibodies to perform Co-IP, OSGEPL1-Myc could not be precipitated with OSGEPL1-FLAG (Figure 1B). Similarly, OSGEPL1-FLAG was not precipitated when using anti-Myc antibodies (Figure 1C). These results suggest that *in vivo*, OSGEPL1-Myc was unable to form a homodimer with OSGEPL1-FLAG. However, when genes encoding a C-terminal Myc-tagged Qri7 (Qri7-Myc) and a C-terminal FLAG-tagged Qri7 (Qri7-FLAG) were co-expressed in HEK293T cells, Qri7-Myc was readily pulled down by Qri7-FLAG, as expected (Figure 1D). Furthermore, hmThrRS-Myc was pulled down by hmThrRS-FLAG, as expected (Figure 1E).

YrdC and OSGEPL1 catalyse two sequential steps during t^6^A modification using TC-AMP as an intermediate. Bacterial TsaC has been shown to interact with TsaD, suggesting formation of a complex that carries out t^6^A formation (13). To explore whether there is an interaction between YrdC and OSGEPL1, we expressed genes encoding OSGEPL1-FLAG and YrdC with a C-terminal HA tag (YrdC-HA). Co-IP analysis of the corresponding proteins showed that YrdC-HA could not be pulled down by OSGEPL1-FLAG and *vice versa* (Figure 1F, G). These results suggest that, in human mitochondria, YrdC does not form a complex with OSGEPL1.

### OSGEPL1 possesses intrinsic ATPase activity

**Figure 2.**
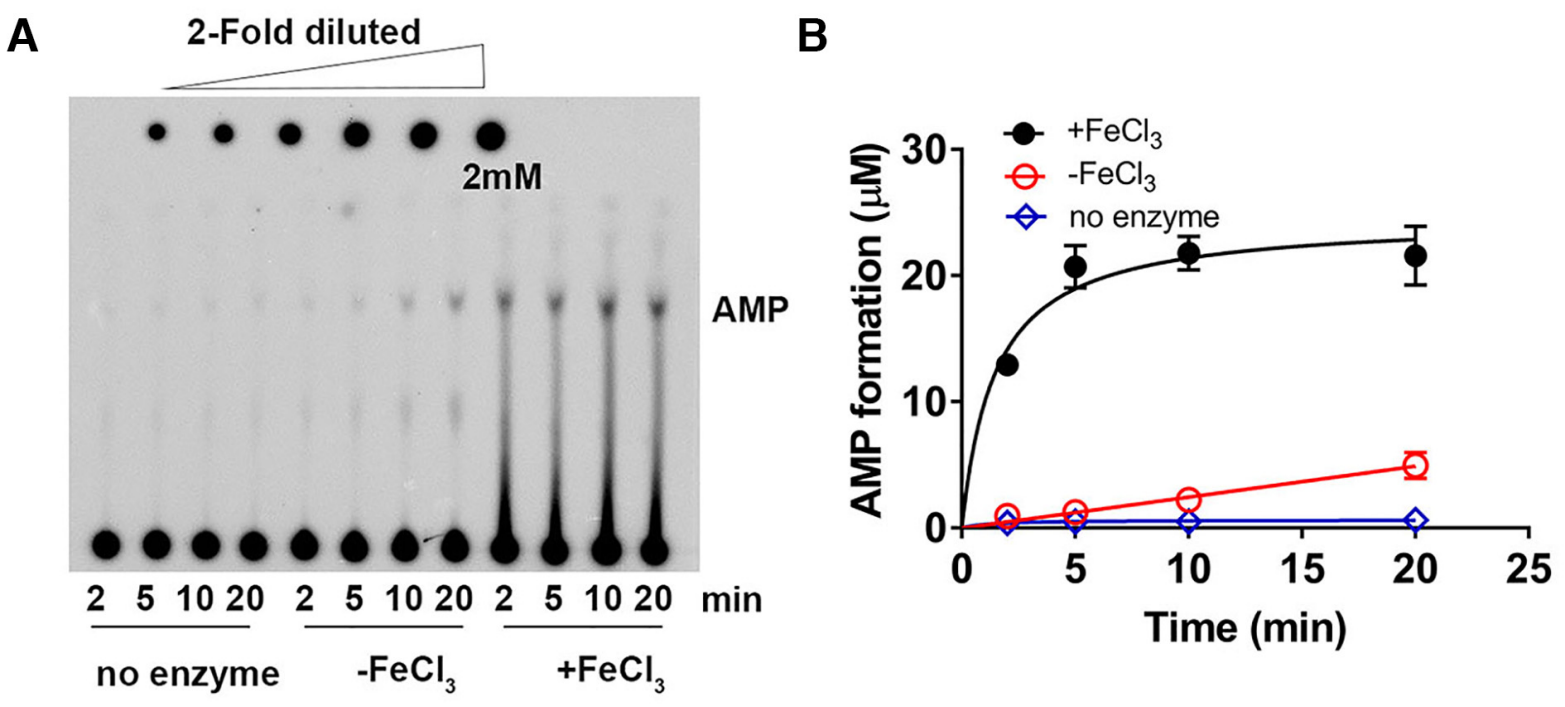
OSGEPL1 possesses ATPase activity. (**A**) A representative TLC image showing AMP formation via ATP hydrolysis in the absence or presence of FeCl_3_. A reaction without adding enzyme was included as a negative control. (**B**) Quantification analysis of AMP formation by OSGEPL1 with (black filled circles) or without (red circles) FeCl_3_. A reaction without addition of OSGEPL1 was included as a control (blue diamonds).

Previous work reported that archaeal Kae1 has intrinsic ATPase activity (41). Indeed, the crystal structure of *Pyrococcus abyssi* Kae1 (PDB 2IVN) revealed the ability to bind ATP through two absolutely conserved His residues in the presence of a chelated Fe^3+^ ion. Simultaneous mutation of these two His residues appears to disrupt ATPase and t^6^A modification activities (10). Herein, we performed ATP hydrolysis assays to investigate whether OSGEPL1 also possesses ATPase activity. The results of TLC analysis (Figure 2A) clearly showed that OSGEPL1 was able to hydrolyse ATP to generate AMP only in the presence of FeCl_3_ (Figure 2B), suggesting that OSGEPL1 indeed has an intrinsic ATPase activity that is dependent on the presence of Fe^3+^.

### The hmtRNA^Thr^ transcript is the best substrate for YrdC/OSGEPL1 *in vitro*

**Figure 3.**
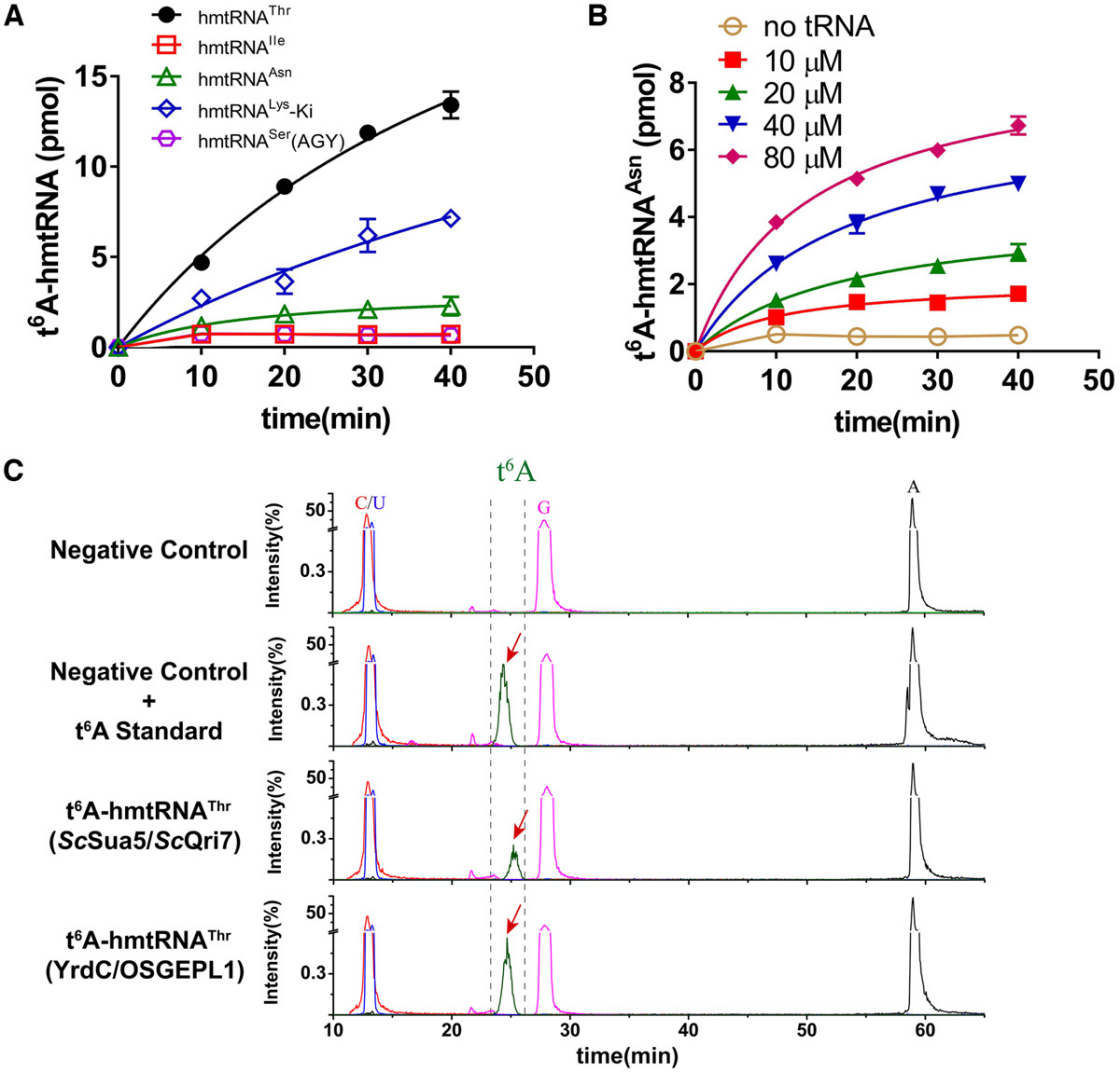
The hmtRNA^Thr^ transcript is the best substrate of YrdC/OSGEPL1 *in vitro*. (**A**) Time-course curves of the modification of five hmtRNAs, hmtRNA^Asn^ (green triangles), hmtRNA^Ile^ (red squares), hmtRNA^Lys^ (blue diamonds), hmtRNA^Ser^(AGY) (pink hexagons) and hmtRNA^Thr^ (black filled circles), by YrdC and OSGEPL1. (**B**) t^6^A modification at increasing concentrations of hmtRNA^Asn^ (10 μM, red filled squares; 20 μM, green filled triangles; 40 μM, blue filled inverted triangles and 80 μM, pink filled diamonds). A control without tRNA addition (no tRNA, orange circles) was included. (**C**) LC–MS/MS analysis of the digestion product of the hmtRNA^Thr^ transcript in the absence and presence of standard t^6^A or modified hmtRNA^Thr^ transcripts by YrdC/OSGEPL1 or Sua5/Qri7.

In human and bovine mitochondria, five tRNAs (hmtRNA^Asn^, hmtRNA^Ile^, hmtRNA^Lys^, hmtRNA^Ser^(AGY) and hmtRNA^Thr^) contain the t^6^A modification (5,29). To reconstitute t^6^A modification activity, their transcripts were obtained by *in vitro* T7 transcription. We transcribed the Ki mutant of hmtRNA^Lys^ (with U50:A64 base substitutions) to prevent misfolding of the WT hmtRNA^Lys^ transcript (51). *In vitro* results showed that hmtRNA^Thr^ and hmtRNA^Lys^-Ki could be t^6^A-modified (Figure 3A). However, the modification efficiency for hmtRNA^Thr^ was higher than for hmtRNA^Lys^-Ki (Figure 3A). The Lys-accepting activity of the hmtRNA^Lys^-Ki transcript was 686 pmol/A_260_ and at the same concentration used for t^6^A modification, hmtRNA^Lys^-Ki was efficiently aminoacylated by hLysRS (Supplementary Figure S3A), suggesting that hmtRNA^Lys^-Ki was at least partially correctly folded. Furthermore, the modification efficiency for hmtRNA^Asn^ was lower than for hmtRNA^Thr^ and hmtRNA^Lys^-Ki. Comparable modification levels were only achieved by adding higher concentrations of hmtRNA^Asn^ (80 μM; Figure 3B). Finally, hmtRNA^Ile^ and hmtRNA^Ser^(AGY) transcripts were not modified at all (Figure 3A). Further experiments showed that failure to modify hmtRNA^Ile^ was due to the presence of G38 (see the results below). For hmtRNA^Ser^(AGY), its Ser-accepting activity was determined to be 985 pmol/*A*_260_. Similarly, it could be readily charged by hmSerRS at the same concentration used for t^6^A modification (Supplementary Figure S3B). These results suggested that hmtRNA^Ser^(AGY) was at least partially correctly folded.

We subsequently performed liquid chromatography with electrospray ionisation tandem mass spectrometry (LC–MS/MS) to validate the t^6^A moiety added in our *in vitro* analysis, and t^6^A was only detected in the hmtRNA^Thr^ transcript upon addition of the standard t^6^A sample. However, t^6^A was readily detected after hmtRNA^Thr^ was incubated with YrdC and OSGEPL1 (Figure 3C). Similarly, as demonstrated in our previous work, t^6^A was present after incubation of hmtRNA^Thr^ with yeast enzymes Sua5 and Qri7 (40).

Taken together, our data showed that, among five t^6^A-modified hmtRNAs, the hmtRNA^Thr^ transcript was the best substrate for *in vitro* YrdC/OSGEPL1 activity determination.

### Nucleotide requirement in the anticodon loop

**Figure 4.**
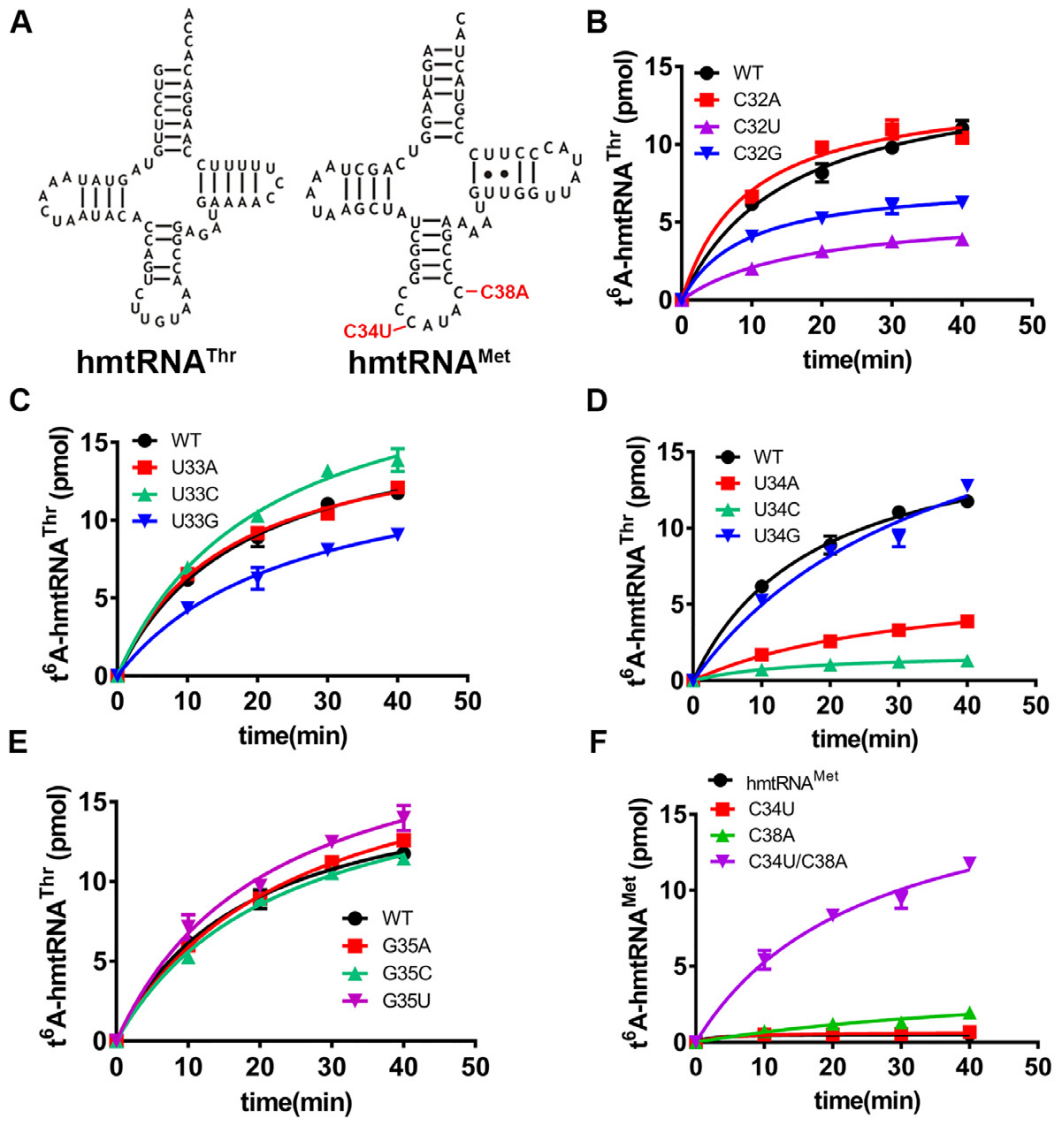
Nucleotide requirements in the anticodon loop. (**A)** Mutagenesis of hmtRNA^Thr^ at the non-Watson-Crick base pair (A29-C41; left), and mutagenesis of hmtRNA^Met^ at the anticodon loop base (C34 and C38; right) are coloured red. (**B**) t^6^A modification levels of hmtRNA^Thr^ (black filled circles) and its mutants C32A (red filled squares), C32U (magenta filled triangles) and C32G (blue filled inverted triangles). (**C**) t^6^A modification levels of hmtRNA^Thr^ (black filled circles) and mutants U33A (red filled squares), U33C (cyanine filled triangles) and U33G (blue filled inverted triangles). (**D**) t^6^A modification levels of hmtRNA^Thr^ (black filled circles) and mutants U34A (red filled squares), U34C (cyanine filled triangles) and U34G (blue filled inverted triangles). (**E**) t^6^A modification levels of hmtRNA^Thr^ (black filled circles) and mutants G35A (red filled squares), G35C (cyanine filled triangles) and G35U (magenta filled inverted triangles). (**F**) t^6^A modification levels of hmtRNA^Met^ (black filled circles) and mutants C34U (red filled squares), C38A (green filled triangles) and C34U/ C38A (magenta filled inverted triangles).

Besides the modification site (A37), only two nucleotides, U36 and A38 in the anticodon loop, have been identified as determinants in t^6^A modification in *Xenopus laevis* oocytes and human mitochondria, respectively (39,40). To more comprehensively explore the key nucleotides, we used hmtRNA^Thr^ as a model and mainly focused on the anticodon loop due to its proximity to the modification site (A37) (Figure 4A, left). U36, A37 and A38 were not further studied because their critical roles have been revealed. C32, U33, U34 and G35 were all mutated to the other three nucleotides, and t^6^A formation was examined. The results showed that modification of C32G and C32U was obviously decreased, more so in the latter case. However, C32A was modified to a comparable level with WT hmtRNA^Thr^. These results suggest that C32 is an important base, but not a determinant in t^6^A formation (Figure 4B). Modification of the three mutants of U33 (U33A, U33C, and U33G) was not significantly affected (Figure 4C), indicating a minimal role in recognition and catalysis. Interesting results were obtained for the U34 mutants; even though t^6^A formation was not affected in U34G, its formation was significantly deceased in the U34A mutant, and t^6^A modification was nearly abolished for U34C (Figure 4D). To understand whether OSGEPL1 was unable to bind U34C efficiently, the *K*_d_ value was determined, and *K*_d_ values of OSGEPL1 with U34C and U34G were comparable with that for WT hmtRNA^Thr^ (Supplementary Table S2). Furthermore, we found that mutation at G35 had no effect on t^6^A modification (Figure 4E). The above results suggest that during t^6^A modification of human mitochondrial tRNAs, C34 is an anti-determinant, and nucleotide requirements in the anticodon loop are ordered (C/A>U>G)^32^-N^33^-(U/G >A)^34^-N^35^-U^36^A^37^A^38^’, where N represents any nucleotides and underlined A is a modified site.

Comparison of modified tRNA species revealed that tRNA^Met^ was readily modified in both *E. coli* and yeast cytoplasm (11,54–56). However, mtRNA^Met^ is not a substrate for t^6^A modification in cow or human mitochondria (5,29). Sequence of its anticodon loop indicated that C34 and C38 appear to be divergent nucleotides, based on our above proposed sequence requirements. To convert hmtRNA^Met^ into a substrate for t^6^A modification, we initially introduced C34U or C38A single-point mutations (Figure 4A, right). However, no significant modification of hmtRNA^Met^-C38A or -C34U mutants was detected, presumably due to the presence of C34 or C38, respectively. Accordingly, after the C34U/C38A double-point mutation was introduced, the hmtRNA^Met^-C34U/C38A mutant was readily modified (Figure 4F). These results also suggest that our proposed sequence requirements in the anticodon loop, namely (C/A>U>G)^32^-N^33^-(U/G>A)^34^-N^35^-U^36^A^37^A^38^, is necessary and sufficient to convert a non-t^6^A substrate into a modified tRNA.

### Neither the yeast cytoplasmic nor the mitochondrial t^6^A modification machinery employs C34 as an anti-determinant

**Figure 5.**
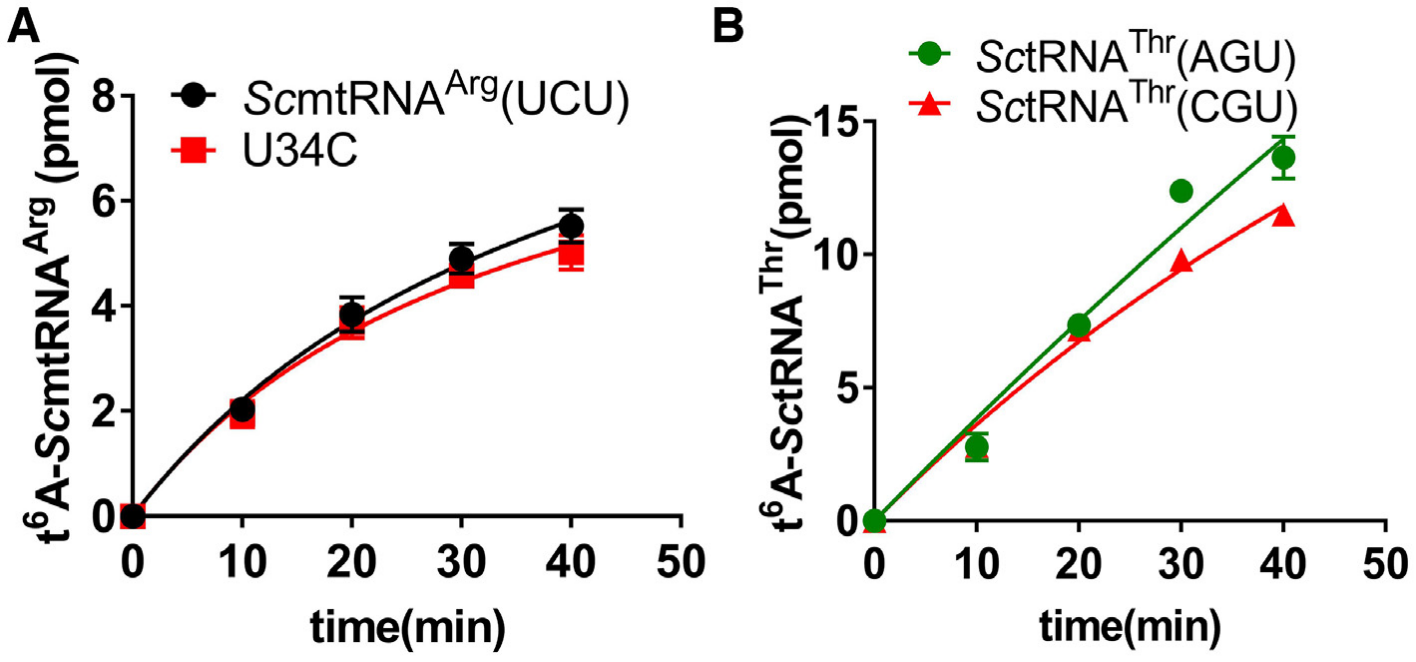
C34 is not an anti-determinant for the yeast t^6^A modification machinery. (**A**) t^6^A modification levels of *Sc*mtRNA^Arg^(UCU) (red filled circles) and *Sc*mtRNA^Arg^(UCU)-U34C (black filled squares) determined with Sua5/Qri7. (**B**) t^6^A modification levels of *Sc*tRNA^Thr^(CGU) (red filled triangles) and *Sc*tRNA^Thr^(AGU) (green filled circles) determined with Sua5/*Sc*KEOPS.

tRNAs with a C34 can be readily t^6^A-modified in *E. coli* and yeast cytoplasm, suggesting C34 is well tolerated in other species. To understand whether yeast mitochondrial t^6^A modification enzymes (Sua5 and Qri7) employ C34 as an anti-determinant, we initially selected *Sc*mtRNA^Arg^(UCU), which has been shown to be t^6^A-modified (57). Both *Sc*mtRNA^Arg^(UCU) and *Sc*mtRNA^Arg^(UCU)-U34C were readily modified with comparable efficiency by Sua5 and Qri7 (Figure 5A). Furthermore, hmtRNA^Thr^ and its U34C mutant were also modified by Sua5 and Qri7 (Supplementary Figure S4A). Additionally, the hmtRNA^Met^ -C38A mutant (with C34) was also t^6^A-modified by Sua5/Qri7 (Supplementary Figure S4B). These results clearly revealed that the yeast mitochondrial enzyme for t^6^A modification does not use C34 as an anti-determinant.

In eukaryotic cytoplasm, t^6^A modification is jointly catalysed by Sua5 and the KEOPS complex. The *S. cerevisiae* KEOPS complex (*Sc*KEOPS) has been purified from yeast cells in our institute, and been shown to catalyse t^6^A modification of *Sc*tRNA^Thr^(AGU) and *Sc*tRNA^Thr^(CGU) (33,58–60). Purified Sua5 and *Sc*KEOPS were assayed for t^6^A modification of *Sc*tRNA^Thr^(AGU) and *Sc*tRNA^Thr^(CGU) transcripts possessing A34 and C34, respectively (Figure 5B). Both tRNAs were clearly modified. In addition, Sua5/*Sc*KEOPS was able to introduce the t^6^A modification in human cytoplasmic (hc) tRNA^Thr^s, hctRNA^Thr^(AGU), hctRNA^Thr^(CGU) and hctRNA^Thr^(UGU), with various bases at position 34 (Supplementary Figure S4C). Finally, Sua5/*Sc*KEOPS was also able to modify hmtRNA^Thr^-U34C (Supplementary Figure S4D).

The above evidence clearly shows that C34 is not an anti-determinant for t^6^A modification by either yeast mitochondrial or cytoplasmic enzymes. Thus, using C34 as an anti-determinant in t^6^A modification by human mitochondrial enzymes was likely a later evolutionary event.

### Sequences of hmtRNA^Ile^ and hmtRNA^Ser^(AGY) are fine-tuned for t^6^A modification

**Figure 6.**
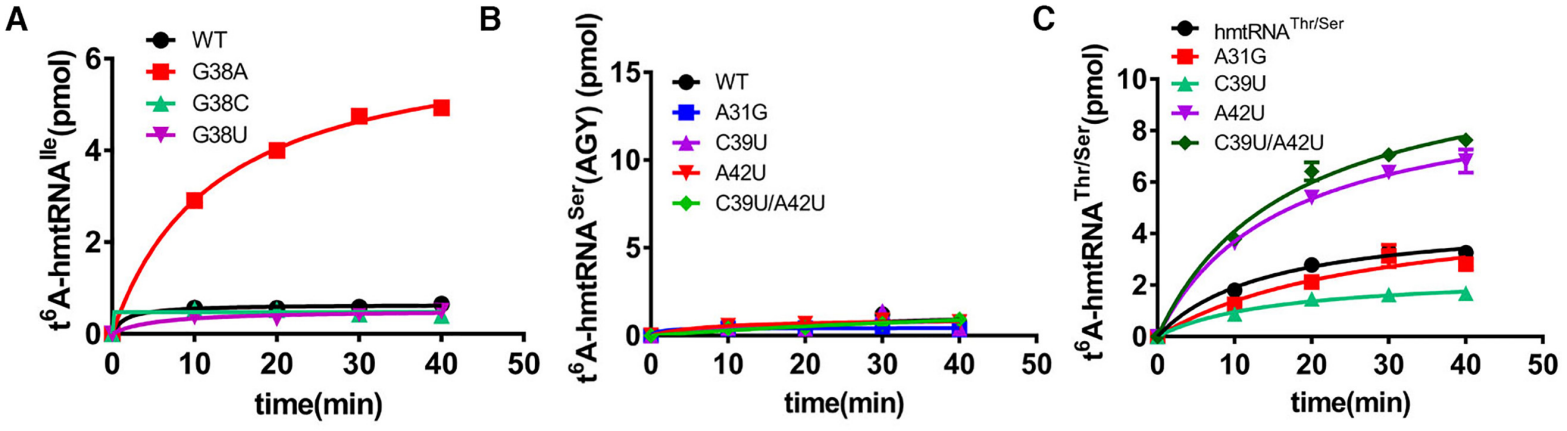
Sequences of hmtRNA^Ile^ and hmtRNA^Ser^(AGY) are fine-tuned for t^6^A modification. (**A**) t^6^A modification levels of hmtRNA^Ile^ (black filled circles) and mutants G38A (red filled squares), G38C (cyanine filled triangles) and G38U (magenta filled inverted triangles) by YrdC/OSGEPL1. (**B**) t^6^A modification levels of hmtRNA^Ser^(AGY) (black filled circles) and mutants A31G (blue filled squares), C39U (magenta filled triangles), A42U (red filled inverted triangles) and C39U/A42U (green filled diamonds) by YrdC/OSGEPL1. (**C**) t^6^A modification levels of hmtRNA^Thr/Ser^ (black filled circles) and mutants A31G (blue filled squares), C39U (cyanine filled triangles), A42U (magenta filled triangles) and C39U/A42U (dark green diamonds) by YrdC/OSGEPL1.

When checking the anticodon loop of the five mitochondrial tRNAs modified with t^6^A, we found that the sequences of hmtRNA^Thr^, hmtRNA^Lys^, hmtRNA^Ser^(AGY) and hmtRNA^Asn^ matched our proposed sequence for t^6^A modification, (C/A>U>G)^32^-N^33^-(U/G>A)^34^-N^35^-U^36^A^37^A^38^, in the anticodon loop. However, hmtRNA^Ile^ obviously deviates from this sequence by the presence of G38 rather than A38. Indeed, the hmtRNA^Ile^ transcript could not be modified by YrdC/OSGEPL1 (Figure 3A). G38A, G38C and G38U mutants of hmtRNA^Ile^ were constructed and transcribed, and only hmtRNA^Ile^-G38A could be obviously modified, further supporting the crucial role of A38 revealed with hmtRNA^Thr^ (Figure 6A). These results suggest that G38 in native hmtRNA^Ile^ may decrease the amount of t^6^A.

hmtRNA^Ser^(AGY) is the most peculiar among all mtRNAs due to its complete lack of D-stem and loop features (Supplementary Figure S5A). Furthermore, two naturally occurring non-Watson-Crick base pairs (A28:A42 and A31:C39) are present in its anticodon stem. However, bovine mitochondrial tRNA^Ser^(AGY) has Watson–Crick A28:U42 and A31:U39 base pairs (5). Furthermore, the WT hmtRNA^Ser^(AGY) transcript could not be modified by YrdC/OSGEPL1 *in vitro* (Figure 3A). To understand whether the two non-Watson–Crick base pairs contribute to its defective modification, A31G, C39U, and the A42U single-point mutation were separately introduced to reform the base pair between G31:C39, A31:U39 and A28:U42 (Supplementary Figure S5B). The double-site mutant tRNA^Ser^(AGY)-C39U/A42U containing A31:39U and 28A:U42 Watson–Crick base pairs in its anticodon stem was also constructed. None of the variants were modified by YrdC/OSGEPL1 (Figure 6B). In a further attempt to achieve t^6^A modification of hmtRNA^Ser^(AGY), we transplanted the whole D-stem and loop features of hmtRNA^Thr^ into hmtRNA^Ser^(AGY). However, the resulting hmtRNA^Ser^(AGY)-D(Thr) chimeric mutant (Supplementary Figure S5C) still could not be modified with t^6^A. Fortunately, upon replacement of the anticodon stem and loop of hmtRNA^Thr^ with that of hmtRNA^Ser^(AGY), the chimeric hmtRNA^Thr/Ser^ mutant (Supplementary Figure S5C) could be modified with t^6^A by YrdC/OSGEPL1 (Figure 6C). Using the chimeric hmtRNA^Thr/Ser^, we further studied the potential effects of two natural non-Watson–Crick base pairs in the anticodon stem of hmtRNA^Ser^(AGY). A31G, C39U and A42U single-site mutants, and the C39U and A42U double-site mutant were separately introduced to reform Watson–Crick base pairs between 31:39 and/or 28:42. The results showed that formation of a Watson–Crick A28:U42 pair markedly increased the level of t^6^A modification; however, restoration of the 31:39 base pair had no positive effect on modification (Figure 6C).

Both G38 of hmtRNA^Ile^ and non-Watson-Crick base pair A28:A42 of hmtRNA^Ser^(AGY) were found to limit the formation of t^6^A, hence we suggest that sequences of hmtRNA^Ile^ and hmtRNA^Ser^(AGY) were likely fine-tuned to optimise t^6^A modification levels during evolution.

### MTS-deleted OSGEPL1 can rescue loss of Kae1 *in vivo*

**Figure 7.**
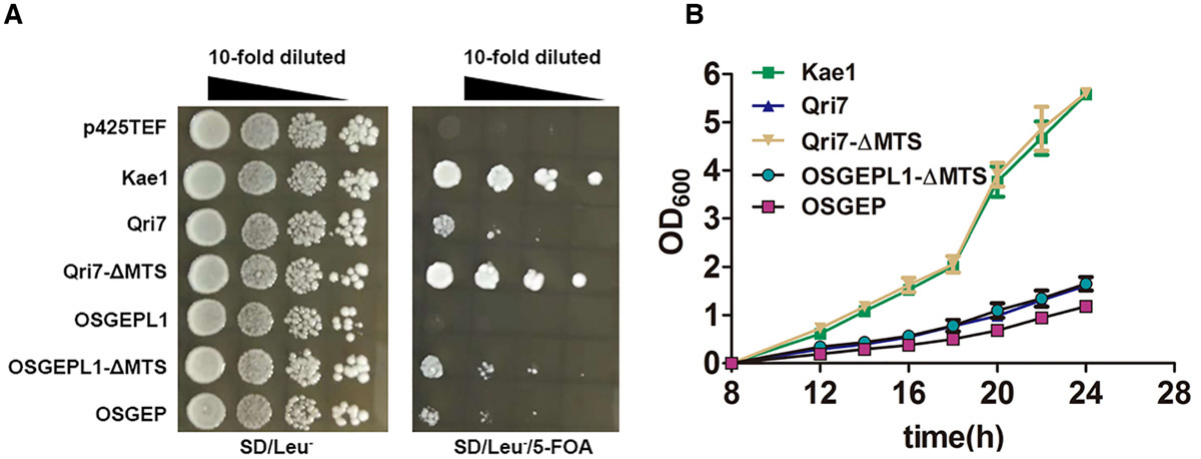
MTS-deleted OSGEPL1 can rescue loss of Kae1 *in vivo*. (**A**) Genes encoding Kae1, Qri7, Qri7-ΔMTS, OSGEPL1, OSGEPL1-ΔMTS and OSGEP were transformed into *Sc*Δ*Kae1*, transformants were spread on SD/Leu^−^ or SD/Leu^−^/5-FOA plates, and the growth phenotype was observed. *Kae1* and p425TEF empty vectors were used as positive and negative controls, respectively. (**B**) Yeast growth curves were determined after 5-FOA selection in SD/Leu^−^ liquid culture at an initial cell density (OD_600_) of 0.03.

MTS-deleted Qri7 (Qri7-ΔMTS) has been shown to efficiently complement the loss of Kae1 in t^6^A modification (25). To perform *in vivo* genetic studies on OSGEPL1, we constructed a *Kae1* gene deletion yeast strain (*Sc*Δ*Kae1*) using homologous recombination. As reported, Qri7-ΔMTS could rescue the loss of the Kae1 gene, as well as WT Kae1. Even the Qri7 precursor was able to support yeast growth despite lower efficiency than Qri7-ΔMTS and Kae1. However, only MTS-deleted OSGEPL1 (OSGEPL1-ΔMTS) but not the mitochondria-localised OSGEPL1 precursor could support cell growth, albeit rather weakly (Figure 7A, B). This rescue is likely due to complementation of t^6^A modification of Kae1 but not its other cellular functions, since Qri7-ΔMTS is not able to restore telomere length maintenance due to loss of Kae1 (58), suggesting that OSGEPL1 can modify cytoplasmic tRNAs, as revealed by *in vitro* studies. We propose that the weak complementation of OSGEPL1-ΔMTS, compared with Qri7-ΔMTS and Kae1, can likely be explained by its inability to modify cytoplasmic C34-containing tRNAs. The human Kae1 homolog OSGEP in *Sc*Δ*Kae1* was also able to complement the loss of Kae1, albeit with lower efficiency.

### OSGEPL1 activity is affected by post-translational modification

**Figure 8.**
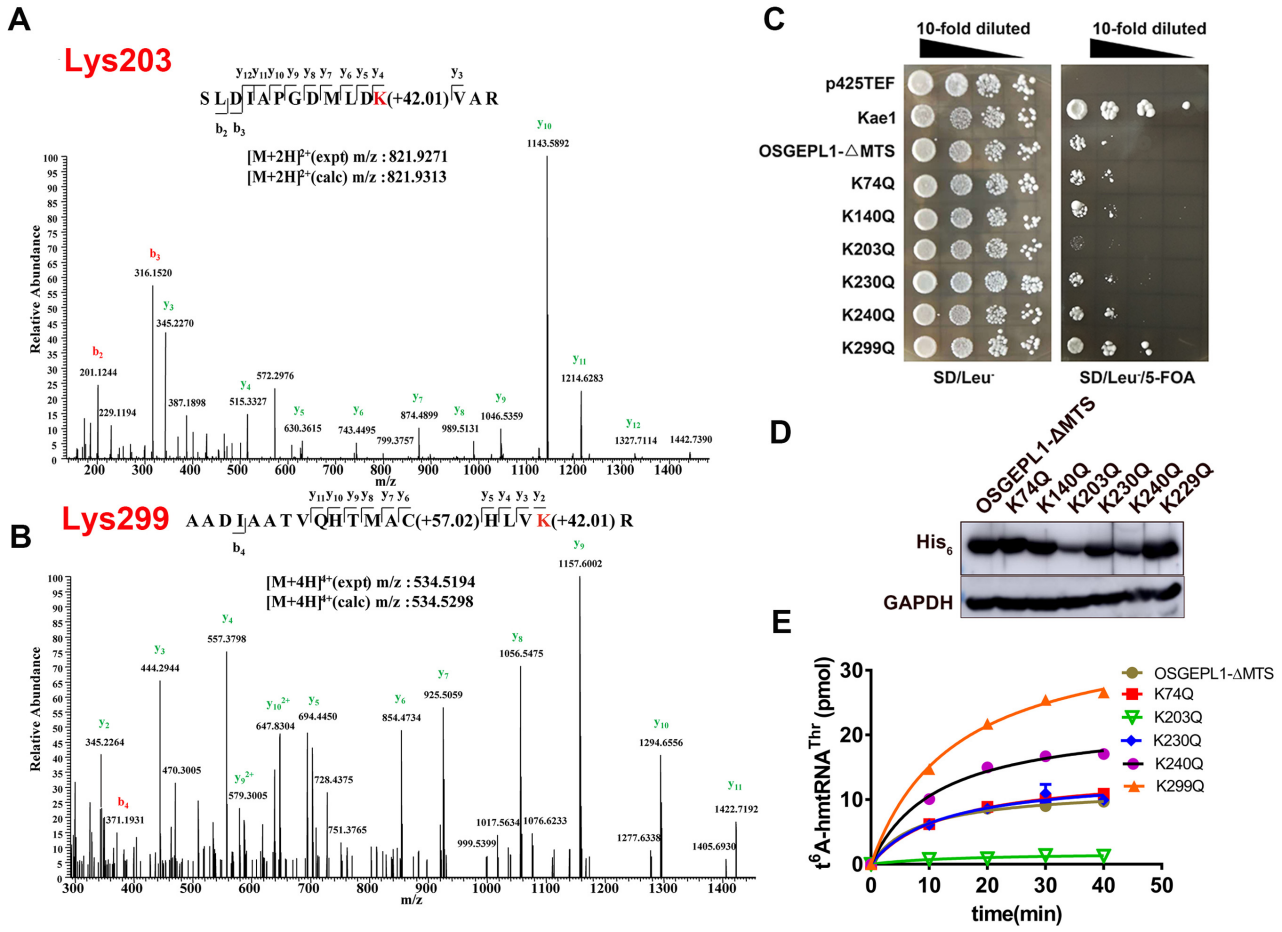
The activity of OSGEPL1 is influenced by post-translational modification. Higher energy collision-induced dissociation (HCD) MS/MS spectra were recorded for (**A**) the [M+2H]^2+^ ion at m/z 821.9313 for human OSGEPL1 peptide SLDIAPGDMLDKVAR harbouring one acetylated site (Lys203), and (**B**) the [M+4H]^4+^ ion at *m*/*z* 534.5298 for the human OSGEPL1 peptide AADIAATVQHTMACHLVKR harbouring one acetylated site (Lys299). Predicted b- and y-type ions (not all) are listed above and below the peptide sequences, respectively. Matched ions are labelled in the spectra. (**C**) Genes encoding Kae1, OSGEPL1-ΔMTS, -K74Q, -K140Q, -K203Q, -K230Q, -K240Q and -K299Q were transformed into *Sc*Δ*Kae1*, transformants were initially cultured in SD/Leu^−^ liquid medium, spread on SD/Leu^−^ or SD/Leu^−^/5-FOA plates, and the growth phenotype was observed on two plates treated with the same 10-fold diluted concentrations (initial OD_600_ = 1.0) as indicated. *Kae1* and p425TEF empty vector were used as positive and negative controls, respectively. (**D**) Western blotting analysis was performed with yeast extracts before 5-FOA selection. (**E**) t^6^A modification activities of OSGEPL1-ΔMTS (brown filled circles), K74Q (red filled squares), K203Q (green filled inverted triangles), K230Q (blue filled diamonds), K240Q (violet filled circles) and K299Q (orange filled triangles).

In order to investigate whether the activity of OSGEPL1 may be affected by post-translational modification, it was purified from mitochondria by Co-IP with anti-FLAG antibodies after expression of OSGEPL1-FLAG, then subsequently analysed by MS. This revealed six acetylation sites; Lys74, Lys140, Lys203, Lys230, Lys240 and Lys299 (Figure 8A, B, Supplementary Figure S6A−D). To probe the potential role of acetylation at each site, we mutated the six Lys residues to Gln to mimic acetylation, resulting in OSGEPL1-K74Q, -K140Q, -K203Q, -K230Q, -K240Q and -K299Q mutants. Genes encoding mature OSGEPL1 and its mutants were transformed into *Sc*Δ*Kae1*, and the results showed that OSGEPL1-K203Q was nearly defective in supporting yeast cell growth, while the growth of yeast cells expressing OSGEPL1-K299Q was most rapid on the SD/Leu^−^/5-FOA plate (Figure 8C). Consistently, yeast growth curves consistently showed the slowest and fastest growth for yeast cells expressing OSGEPL1-K203Q and -K299Q, respectively (Supplementary Figure S6E). Thus, it seems that acetylation of Lys203 might turn off the activity of OSGEPL1.

Western blotting was performed for steady state analysis of these mutants *in vivo*. Compared with OSGEPL1-ΔMTS, only -K203Q was characterised by a lower protein level in yeast, indicating that the stability of this mutant was decreased (Figure 8D). We purified mature OSGEPL1 and it mutants (only -K140Q could not be purified due to aggregation) from *E. coli* and tested the effect of acetylation of the five Lys residues on t^6^A modification activity. Consistent with the failure in complementation experiments, the activity of OSGEPL1-K203Q was lost (Figure 8E). Surprisingly, the activity of OSGEPL1-K299Q was significantly increased, consistent with the growth of yeast expressing OSGEPL1-K299Q (Figure 8E, Supplementary Figure S6E). However, the activities of the other three mutants (OSGEPL1-K74Q, -K230Q and -K240Q) were not significantly different from the WT enzyme. We further compared the binding affinity parameters between hmtRNA^Thr^ with OSGEPL1-ΔMTS, -K203Q and -K299Q, and OSGEPL1-K203Q did indeed exhibit a higher *K*_d_ value (∼2-fold) than OSGEPL1-ΔMTS, while that of OSGEPL1-K299Q was not significantly altered (Supplementary Table S3). The above results indicate nearly abolished or enhanced activity *in vitro* and *in vivo* following acetylation at Lys203 or Lys299, respectively. However, acetylation at other sites had only a slight effect on enzyme activity. Thus, we focused on Lys203 and Lys299 in more detail in subsequent experiments.

Primary sequence alignment showed that Lys203 is highly conserved (Supplementary Figure S7A). Structural analysis based on the yeast Qri7 crystal structure (PDB 3WUH) (28) showed that Lys204 (the Lys203 counterpart) is located at the bottleneck location of the U-shaped cavity of Qri7, and is surface-exposed. The AMP moiety of TC-AMP is located at the bottom of the cavity, implying an active site location (Supplementary Figure S7B). Based on this information, we suggest that Lys203 is likely to bind tRNA during catalysis. We further mutated Lys203 to Ala (no charge), Arg (positive charge) and Glu (negative charge). *In vitro* assays showed that the activities of all mutants were significantly deceased (Supplementary Figure S7C), and all were unable to efficiently supplement loss of Kae1 *in vivo* (Supplementary Figure S7D). Because the three mutants at Lys203 yielded the same amount of OSGEPL1, their decreased activity showed the importance of this residue to the enzyme function (Supplementary Figure S7E). The *K*_d_ values of OSGEPL1-K203R and -K203E were altered slightly, while that of K203A was enhanced by more than 4-fold (29 μM) compared with that of OSGEPL1 (6.7 μM; Supplementary Table S3). These results suggest that Lys203 appears to be a crucial residue for OSGEPL1 activity by acting as a tRNA binding element.

The significantly increased activity of K299Q *in vitro* and *in vivo* was unexpected. Lys299 is a non-conservative residue (Supplementary Figure S7A), located far from the putative active site and tRNA binding surface, and its counterpart is Asn301, Glu234 and Ile249 in yeast Qri7, yeast Kae1, and *E. coli* TsaD, respectively. Based on the Qri7 structure, Asn301 is located on an α-helix at the edge of the molecule and is solvent-exposed (Supplementary Figure S8A). As described above for Lys203, the OSGEPL1-K299A, -K299R and -K299Q mutants were constructed and purified. Interestingly, the activity of OSGEPL1-K299A was greatly increased, whereas the activities of OSGEPL1-K299R and -K299E were nearly identical with that of WT OSGEPL1 (Supplementary Figure S8B). Consistently, OSGEPL1-K299A supported faster growth than the other mutants in yeast complementation assays (Supplementary Figure S8C). Furthermore, except for OSGEPL1-K299R, the steady-state quantities of the other mutant proteins were significantly higher than that of the WT enzyme (Supplementary Figure S8D). However, the amount of OSGEPL1-K299R was lower than that of the native enzyme, and consistently, this mutant was less efficient at supporting yeast growth (Supplementary Figure S8C). These results suggest that acetylation or mutation at Lys299 altered the protein structure, possibly by subtly changing the conformation. Some conformational changes, such as those induced by acetylation or Ala replacement, may stimulate the activity of OSGEPL1 *in vitro* and *in vivo*.

Taken together, the above combination of acetylation and site-directed mutagenesis studies suggest that acetylation of OSGEPL1, at least at positions Lys203 and Lys299, had a direct inhibitory or stimulatory effect on the t^6^A modification activity of OSGEPL1. The positively charged Lys203 appears to be a tRNA binding element in OSGEPL1, while Lys299 controls subtle conformational changes in OSGEPL1 to elevate enzyme activity. It is notable that both OSGEPL1-K299Q and -K299A variants were more active for t^6^A modification of human mitochondrial tRNA substrates than the WT enzyme.

## DISCUSSION

In bacteria (e.g. *E. coli*, *Salmonella typhimurium* and *Thermotoga maritima*), TsaD alone is unable to bind tRNA, and catalyses the transfer of the TC-moiety. Interaction between TsaD and TsaB is required for tRNA binding and catalysis (14,15,61,62). In archaea (e.g. *P. abysii*) and eukaryotic cytoplasm (e.g. *S. cerevisiae* and *Homo sapiens*), Kae1 is one of the components of the KEOPS complex, and Bud32/PRPK must interact with Pcc1 (in archaea and yeast) or LAGE3 (in human cytoplasm), which performs a similar role to TsaB in the quaternary structure and t^6^A generation (25). Even the minimal yeast mitochondrial Qri7 must form a homodimer to be catalytically active (25). Based on the Qri7 structure, one of the Qri7 subunits in the homodimer functions in the same manner as TsaB in the TsaB/TsaD complex or Pcc1 in the Kea1/Pcc1 complex (25). Indeed, ignoring the catalytic function, TsaB is a paralog of TsaD and Qri7 (63). Interestingly, we found that OSGEPL1 is monomeric *in vitro* and *in vivo*, suggesting that a single OSGEPL1 molecule is able to bind tRNA, perform multiple turnovers, and transfer the TC-moiety from TC-AMP to A37 of tRNA. Therefore, human OSGEPL1 is unique in terms of acting in monomeric form. Whether a co-factor, functionally homologous to TsaB or Pcc1, exists in human mitochondria to enhance catalytic efficiency during t^6^A formation is unclear, and needs further exploration. In addition, TsaC has been reported to interact with both TsaB and TsaD, suggesting that bacterial t^6^A generation requires an intact complex (13). However, our results showed that human YrdC does not interact with OSGEPL1. Accordingly, how YrdC releases TC-AMP and how OSGEPL1 captures it efficiently are open questions to be addressed. It is possible that OSGEPL1 engages in a transient interaction with YrdC after synthesis of TC-AMP to prevent its diffusion into solution.

Little is known about the biogenesis and the detailed molecular mechanism of t^6^A formation, especially the tRNA recognition mechanism. A previous *in vivo* study using *X. laevis* oocytes revealed that only U36 and A37 are absolutely required for t^6^A formation; positions 34 and 35 are neutral and tolerate any nucleotides, and one mismatch in the anticodon stem has no effect on modification efficiency (39). However, our recent study on the pathogenic hmtRNA^Thr^ mutant demonstrated that A38 is a determinant for t^6^A modification, at least for mitochondrial t^6^A formation (40). In the present study, we elucidated the tRNA recognition mechanism for the anti-codon loop during modification. The results clearly revealed that, for human mitochondrial t^6^A modification, (C/A>U>G)^32^-N^33^-(U/G>A)^34^-N^35^-U^36^A^37^A^38^ is the required element in the anticodon loop. Specifically, tRNAs with a C34 were unable to be modified by YrdC/OSGEPL1, as illustrated by the failed modification for hmtRNA^Thr^-U34C, hmtRNA^Met^, and hmtRNA^Met^-C38A. These results are distinct from these obtained in the *X. laevis* oocyte study (39). We also revealed that neither yeast cytoplasmic Sua5/KEOPS nor mitochondrial Sua5/Qri7 employs C34 as an anti-determinant, suggesting that the human mitochondrial t^6^A modification machinery is the only exception that utilises C34 as an anti-determinant. Indeed, tRNAs with C34 from *E. coli*, yeast and human, including *E. coli* tRNA^Met^, tRNA^Arg^(CCU), tRNA^Thr^(CGU), *Sc*tRNA^Thr^(CGU) and human cytoplasmic tRNA^Lys^(CUU), all readily undergo t^6^A modification (11,54–56).

Despite the detection of various levels of t^6^A modification in hmtRNA^Thr^, hmtRNA^Lys^, hmtRNA^Ile^, hmtRNA^Ser^(AGY) and hmtRNA^Asn^ (29), t^6^A-modification determination showed that the hmtRNA^Thr^ transcript was the best substrate *in vitro*. Regarding hmtRNA^Ile^, we further confirmed that the presence of the natural G38 nucleotide precludes its modification; hmtRNA^Ile^-G38A was obviously and efficiently modified. These results are also consistent with those from another group showing that t^6^A modification was elevated following G38A mutation (29). Interestingly, G38A is a pathogenic point mutation (64), possibly due to increased t^6^A levels, suggesting that human mitochondrial tRNA has to accurately balance the level of t^6^A modification, and a decrease or increase in modification level appears to pose a threat to mitochondrial function. Furthermore, lack of t^6^A modification for hmtRNA^Ser^(AGY) is not due to the absence of D-stem or loop features, because incorporation of these features of hmtRNA^Thr^ failed to restore its modification. Furthermore, constructing the chimeric hmtRNA^Thr/Ser^ variant showed that the presence of two mismatches in the anticodon stem negatively affected its modification, and the formation of a Watson-Crick base pair at A28:A42 but not A31:C39 improved modification. We suggest that in hmtRNA^Ser^(AGY), the two mismatches fine-tune the t^6^A modification level, similar to the effect observed for G38 in hmtRNA^Ile^. These observations suggest that the sequences of hmtRNA^Ile^ and hmtRNA^Ser^(AGY) are fine-tuned for appropriate level of t^6^A modification.

Our results also clearly indicate that human mitochondrial t^6^A levels are influenced by post-translational acetylation modification of OSGEPL1. Acetylation at various sites of OSGEPL1 does indeed influence the structure and/or function of OSGEPL *in vitro* and *in vivo*; in the majority of cases, acetylation of mitochondrial enzymes has an inhibitory effect (65), consistent with the decreased activity after acetylation at Lys203 of OSGEPL1. Herein, we deduced its spatial location, and the mutagenesis results implicate Lys203 as a key residue for tRNA binding. Thus, acetylation at position Lys203 may disturb proper tRNA binding. Surprisingly, our results showed that acetylation at Lys299 of OSGEPL1 had a stimulatory effect, and further analysis showed that mutation of Lys299 to Ala induced a similar phenotype. We predict that Lys299 is not involved in substrate binding or catalysis, but rather controls the enzyme structure by finely regulating the local conformation. These two lines of evidence indicate that a single type of modification at different sites in a single protein can have a profound effect.

Approximately 63% of mitochondrially localized proteins contain acetylation sites (65). Mitochondrial acetylation is thought to be non-enzymatically introduced by reactive lysine residues (65) and acetyl-CoA, and removed by the enzymatic activity of NAD^+^-dependent deacetylase sirtuin 3 (SIRT3) (66). We propose that addition and removal of acetylation of OSGEPL1 is a rapid and efficient way to dynamically regulate OSGEPL1 function, thereby affecting mitochondrial t^6^A level, and subsequently enhancing the fidelity of mitochondrial protein synthesis for timely adaptation to various cellular conditions and stresses. This mitochondrial tRNA t^6^A-modification-mediated crosstalk is probably crucial for mitochondrial function and cell survival. Indeed, it has been reported that t^6^A abundance may be altered when cells are subjected to H_2_O_2_-induced stress (67). This type of response to such stress may, at least in part, be dynamically regulated by post-translational protein modification.

## Supplementary Material

gkaa093_Supplemental_File
