# Supplementary material for: Molecular basis for t6A modification in human mitochondria
Source: Nucleic Acids Res. 2020 Feb 12;48(6):3181–94. doi: 10.1093/nar/gkaa093 (PMC7102964; doi:10.1093/nar/gkaa093)
Supplement: gkaa093_Supplemental_File [file gkaa093_supplemental_file.docx]

**SUPPLEMENTARY FIGURES**


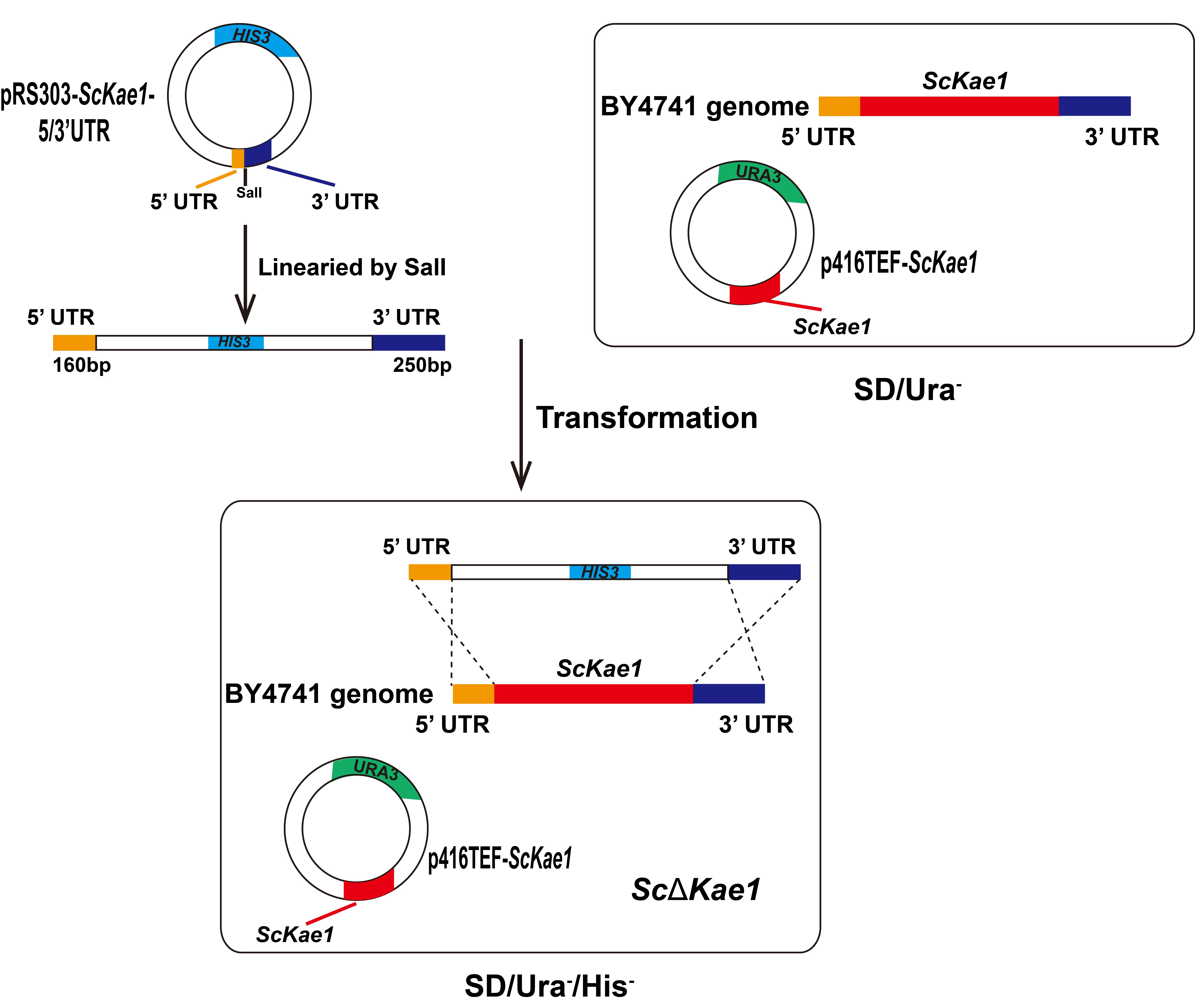


**Supplementary Figure 1. Construction of *Sc*Δ*Kae1* strain**


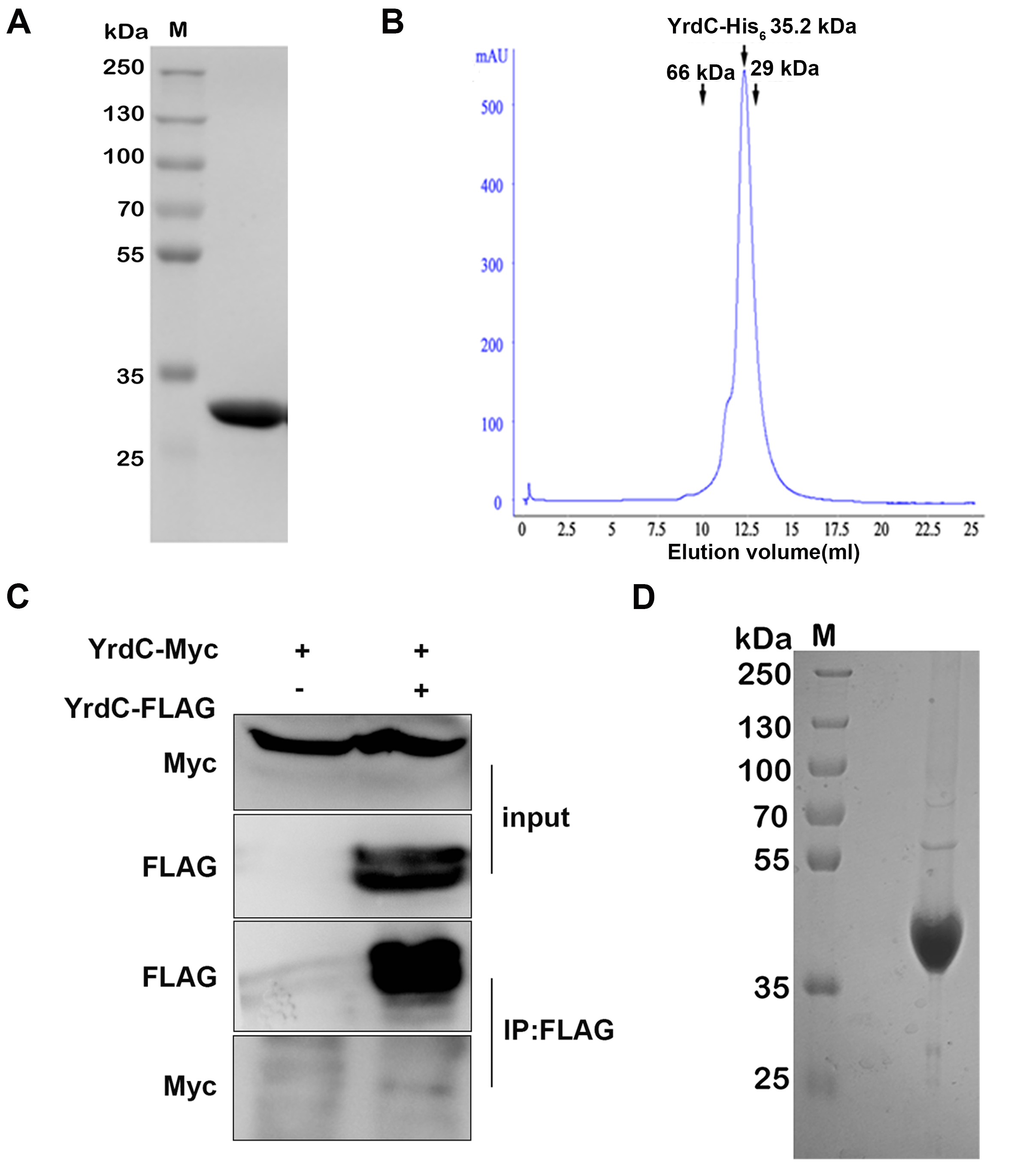


**Supplementary Figure 2. YrdC is a monomer**

(**A**) SDS-PAGE analysis of purified YrdC from *E. coli* with protein markers indicated on the left. (**B**) Gel filtration analysis of YrdC. Elution volumes of bovine serum albumin (66 kDa) and carbonic anhydrase (29 kDa) are also indicated by arrows. (**C**) Genes encoding YrdC-FLAG and YrdC-Myc were co-expressed in HEK293T cells, and YrdC-Myc could not be pulled down by YrdC-FLAG in a Co-IP assay. (**D**) SDS-PAGE analysis of purified mature OSGEPL1 from *E. coli*.


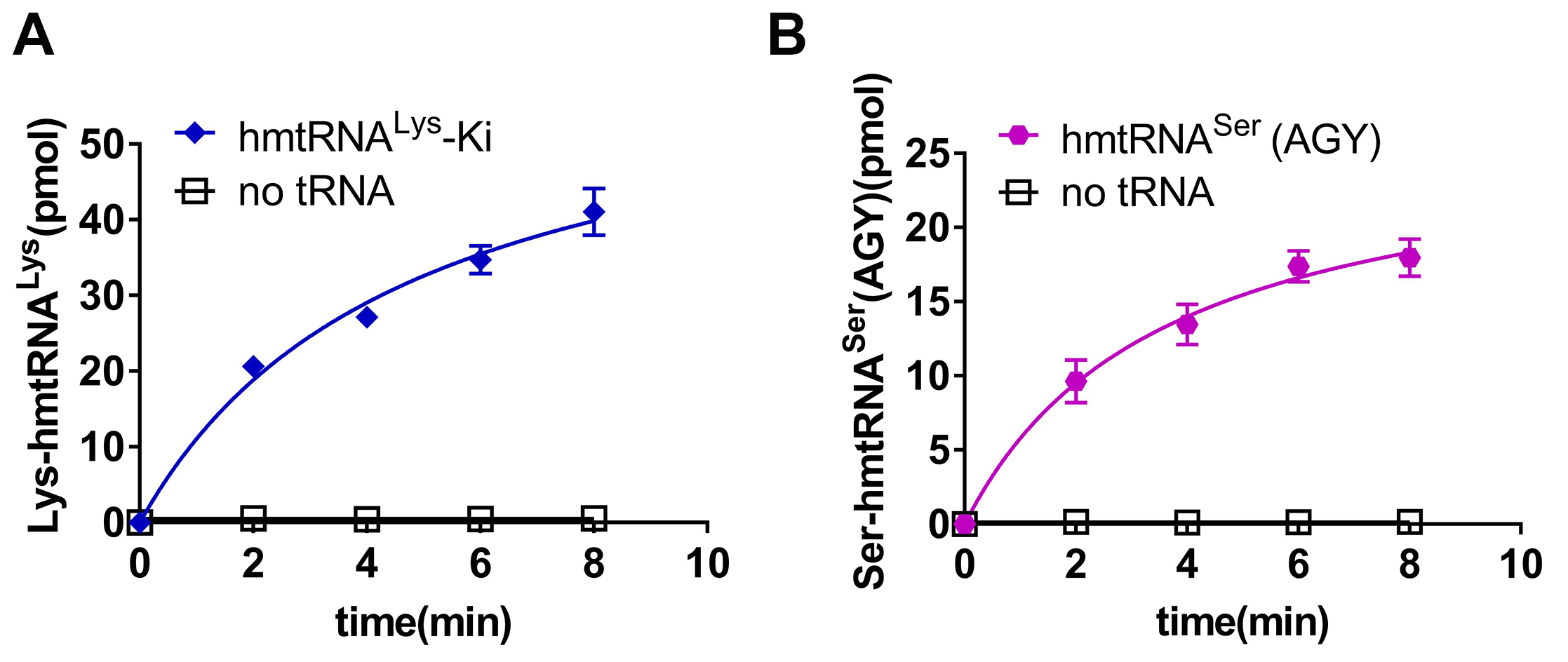


**Supplementary Figure 3. Aminoacylation time-course curves for hmtRNA^Lys^-Ki and hmtRNA^Ser^(AGY)**

Aminoacylation of hmtRNA^Lys^-Ki (blue diamonds) by hLysRS (**A**) or hmtRNA^Ser^(AGY) (pink hexagons) by hmSerRS (**B**). Controls without tRNA addition (no tRNA) (black squares) were included.


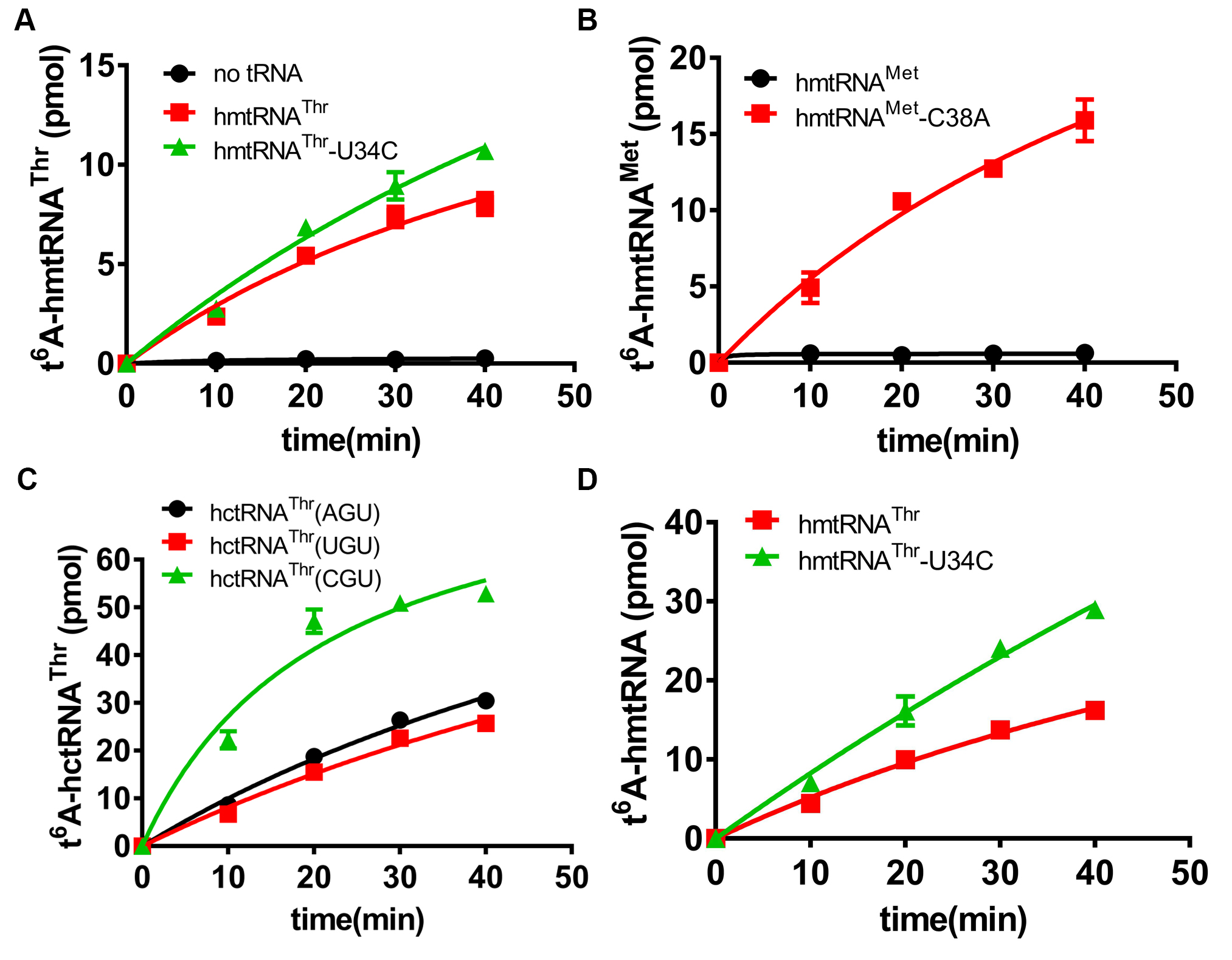


**Supplementary Figure 4. C34 is not an anti-determinant for yeast cytoplasmic or mitochondrial t^6^A modification machinery**

(**A**) t^6^A modification levels of hmtRNA^Thr^ (red filled squares) and hmtRNA^Thr^-U34C (green filled triangles) determined with Sua5/Qri7. (**B**) t^6^A modification levels of hmtRNA^Met^ (black filled circles) and hmtRNA^Met^-C38A (red filled squares) determined with Sua5/Qri7. Reactions with no tRNA added were included as negative controls in (A) and (B). (**C**) t^6^A modification levels of hctRNA^Thr^(AGU) (black filled circles), hctRNA^Thr^(UGU) (red filled squares) and hctRNA^Thr^(CGU) (green filled triangles) determined with Sua5/*Sc*KEOPS. (**D**) t^6^A modification levels of hmtRNA (red filled squares), hmtRNA^Thr^-U34C (green filled triangles), determined with Sua5/*Sc*KEOPS.


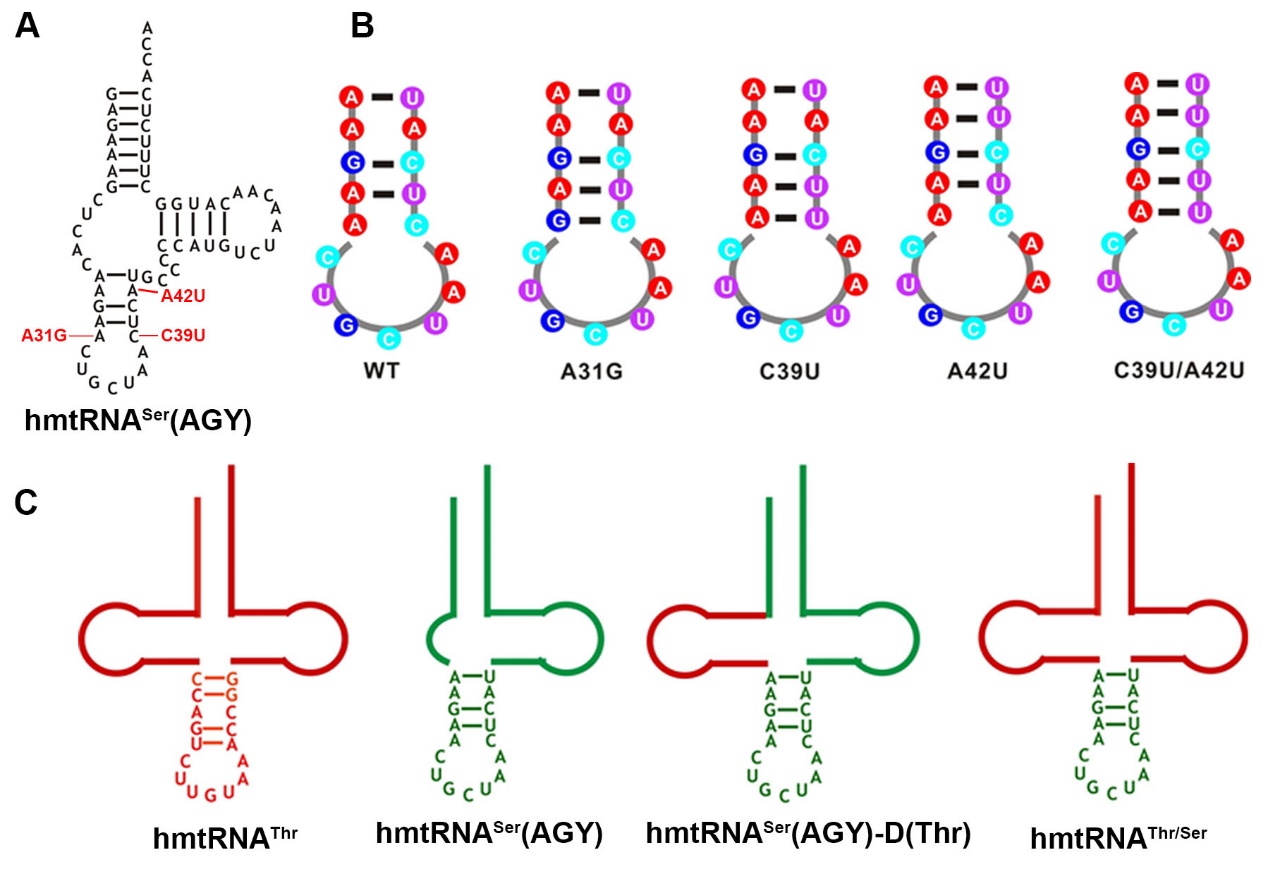


**Supplementary Figure 5.** **Schema showing mutagenesis of hmtRNA^Ser^(AGY)**

(**A**) Mutagenesis of hmtRNA^Ser^(AGY) at non-Watson-Crick base pairs A31:C39 and A28:A42. (**B**) A31G, C39U, A42U and C39U/A42U mutations were introduced in the anticodon stem. (**C**) Construction of hmtRNA^Ser^(AGY)-D(Thr) and hmtRNA^Thr/Ser^ based on hmtRNA^Thr^ and hmtRNA^Ser^(AGY).

**
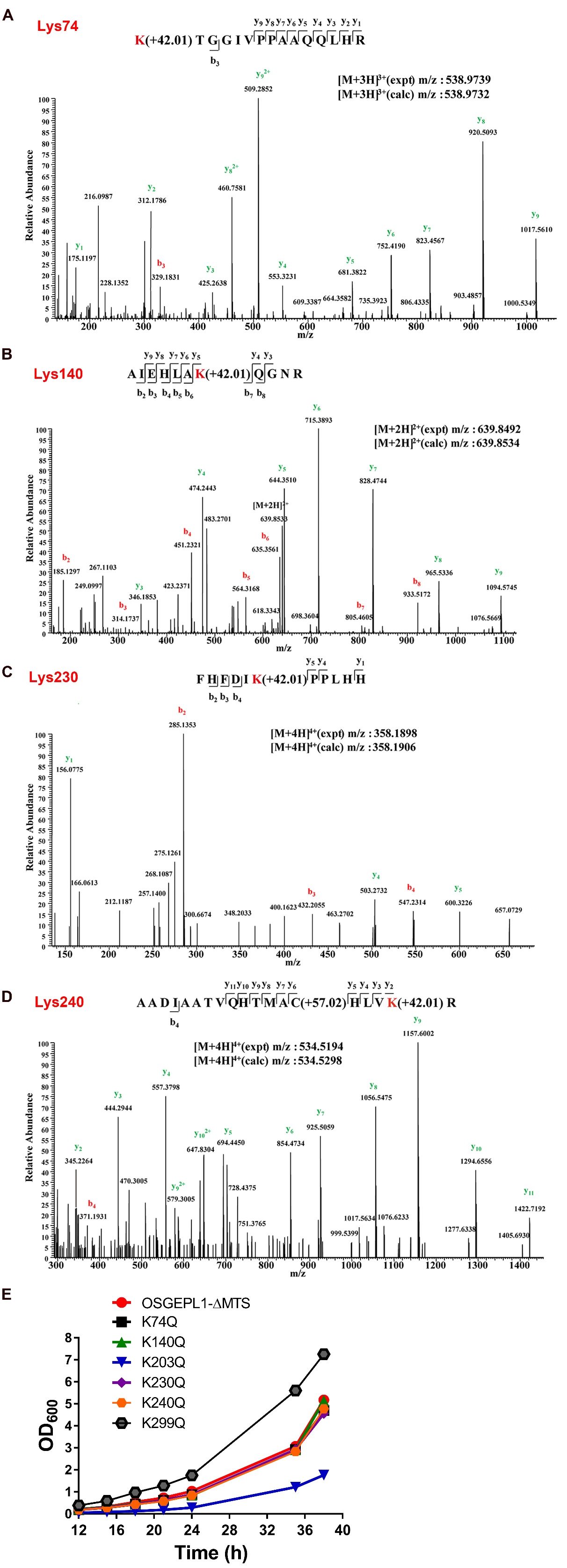
**


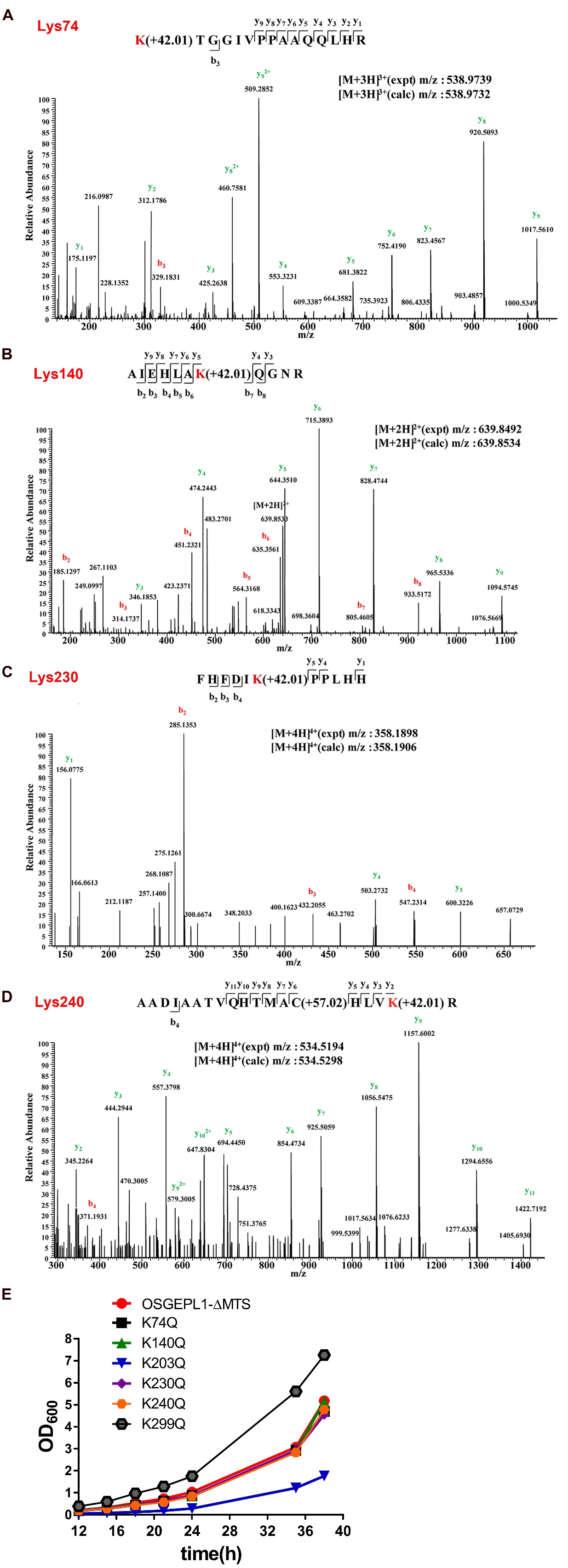


**Supplementary Figure 6. Identification of OSGEPL1 acetylation sites by MS**

Higher energy collision-induced dissociation (HCD) MS/MS spectra showing (**A**) the [M+3H]^3+^ ion at m/z 538.9732 for human OSGEPL1 peptide KTGGIVPPAAQQLHR harboring one acetylated site (Lys74); (**B**) the [M+4H]^4+^ ion at m/z 375.4521 for human OSGEPL1 peptide KPFIPIHHMEAH harbouring one acetylated site (Lys140); (**C**) the [M+2H]^2+^ ion at m/z 639.8534 for human OSGEPL1 peptide AIEHLAKQGNR harboring one acetylated site (Lys230); (**D**) the [M+4H]^4+^ ion at m/z 358.1906 for human OSGEPL1 peptide FHFDIKPPLHH harboring one acetylated site (Lys240). Predicted b- and y-type ions (not all) are listed above and below the peptide sequences, respectively. Matched ions are labelled in the spectra. (**E**) Yeast growth curves determined after 5-FOA selection in SD/Leu^-^ liquid culture with the same initial cell density (OD_600_ = 0.03).


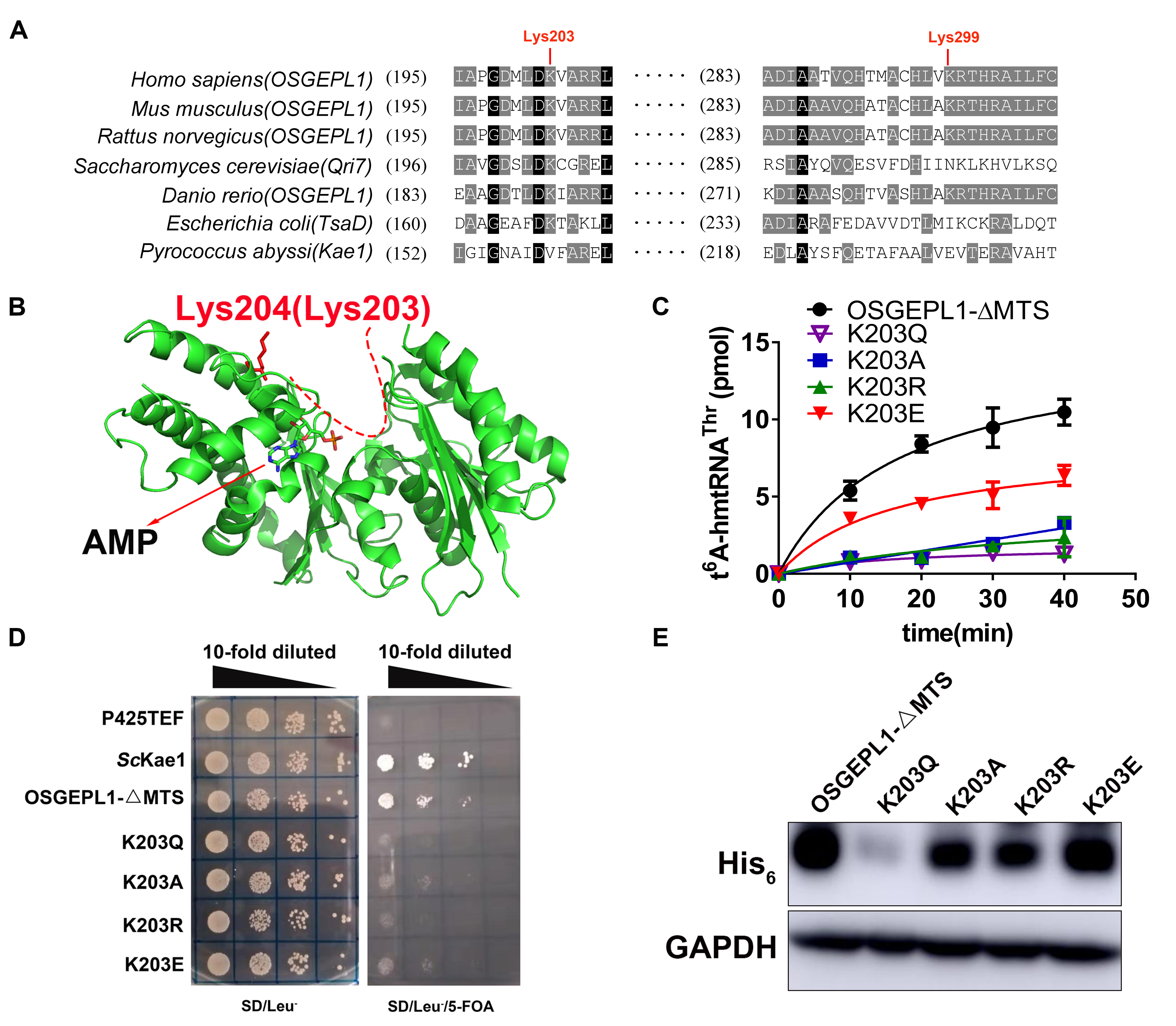


**Supplementary Figure 7. Role of OSGEPL1 residue Lys203**

**(A)** Sequence alignment of TsaD/Qri7/Kae1/OSGEPL1 from various species (*Homo sapiens*, *Mus norvegicus*, *Rattus norvegicus, Saccharomyces cerevisiae*, *Escherichia coli*, *and Pyrococcus abyssi*) in regions covering Lys203 and Lys299*.* (**B**) Structural analysis based on the yeast Qri7 crystal structure (PDB 3WUH) showing that Lys204 is located at the bottleneck location of the U-shaped cavity of Qri7 (corresponding residues in OSGEPL1 are shown in brackets). The U-shaped dotted line represents the putative tRNA binding cavity. (**C**) t^6^A modification activities of OSGEPL1-ΔMTS (black filled circles), -K203Q (dark purple inverted triangles), -K203A (blue filled squares), -K203R (dark green triangles) and -K203E (red filled inverted triangles). (**D**) Genes encoding Kae1, OSGEPL1-ΔMTS, -K203Q, -K203A, -K203R and -K203E were transformed into *Sc*Δ*Kae1*, transformants were cultured in SD/Leu^-^ liquid medium, spread on SD/Leu^-^ or SD/Leu^-^/5-FOA plates, and the growth phenotype was observed on two plates with the same 10-fold diluted concentrations (initial OD_600_ = 1.0) as indicated. *Kae1* and p425TEF empty vectors were used as positive and negative controls, respectively. (**E**) Western blotting analysis was performed with yeast extracts before 5-FOA selection.


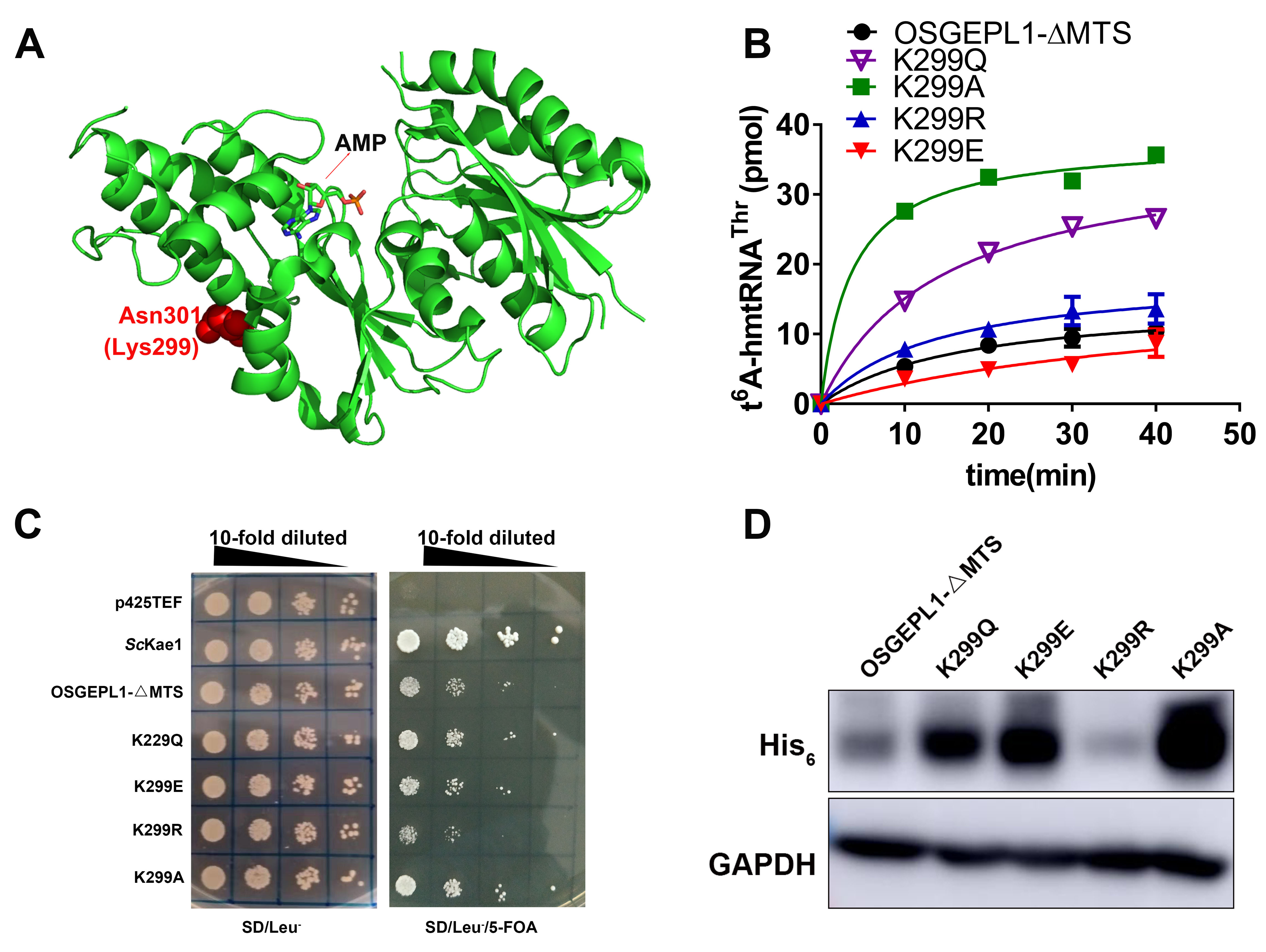


**Supplementary Figure 8. Role of OSGEPL1 residue Lys299**

(**A**) Structural analysis based on the yeast Qri7 crystal structure (PDB 3WUH) showing that Asn301 (the Lys299 counterpart in OSGEPL1) is located far from the putative active site and tRNA binding surface. (**B**) t^6^A modification activities of OSGEPL1-ΔMTS (black filled circles), -K299Q (dark purple inverted triangles), -K299A (green filled squares), -K299R (blue filled triangles) and K299E (red filled inverted triangles). (**C**) Genes encoding Kae1, OSGEPL1-ΔMTS, -K299Q, -K299A, -K299R and -K299E were transformed into *Sc*Δ*Kae1*, transformants were cultured in SD/Leu^-^ liquid medium, spread on SD/Leu^-^ or SD/Leu^-^/5-FOA plates, and the growth phenotype was observed on two plates with the same 10-fold diluted concentrations (initial OD_600_ = 1.0) as indicated. *Kae1* and p425TEF empty vectors were used as positive and negative controls, respectively. (**D**) Western blotting analysis was performed with yeast extracts before 5-FOA selection.

| **Supplementary Table 1. Primers for cloning and tRNA transcription** | |
| --- | --- |
| Name | Primer (5’ to 3’) (restriction sites italic) |
| Qri7-F | cgc*ggatcc*atgatatcaataaaaggaac |
| Qri7-R | ccg*ctcgag*tagctggtccgttctccaac |
| mature-Qri7-F | cgc*ggatcc*atgaaagttttagccattgagacttc |
| YrdC-F | cgc*ggatcc*atgctaatcttgactaagac |
| YrdC-R | ccg*ctcgag*tatctccatttttaattgtg |
| OSGEPL1-F | cgc*ggatcc*atgctaatcttgactaagac |
| OSGEPL1-R | ccg*ctcgag*tatctccatttttaattgtg |
| mature-OSGEPL1-F | cgc*ggatcc*atgcttcataaaatagtattggg |
| mature-hmSerRS-F | cgc*catatg*actacagagaaacgaaaccgga |
| hmSerRS-R | cgc*gcggccgc*ttagcttacagcaggctggccag |
| Kae1-F | cgc*actagt*atggtcaacttgaacact |
| Kae1-R | cgc*gtcgac*atcacgccaggctgcgta |
| OSGEP-F | cgc*actagt*atgccggcggtgctgggt |
| OSGEP-R | cgc*gtcgac*gtccctccaggtcacctc |
| hmtRNA^Thr^-temp-R | tggtgtccttggaaaaaggt |
| hmtRNA^Ile^-temp-R | tggtagaaataagggggttt |
| hmtRNA^Asn^-temp-R | taagcaccctaatcaactgg |
| hmtRNA^Lys^-Ki-temp-R | tggtcactgtaaagaggtgt |
| hmtRNA^Ser^(AGY)-temp-R | tggtgagaaagccatgttgt |
| hctRNA^Thr^(CGT)-temp-R | tggaggcacggacggggt |
| hctRNA^Thr^(AGT)-temp-R | tggaggccccgctgggattc |
| hctRNA^Thr^(TGT)-temp-R | tggaggccccagcgagattt |
| *Sc*tRNA^Thr^(AGU)-temp-R | tggtgcttccaatcggattt |
| *Sc*tRNA^Thr^(CGU)-temp-R | ggtgccctctgtgggaattg |
| *Sc*mtRNA^Arg^(UCU)-temp-R | tggtactctctccatgattt |

| Supplementary Table 2. *K*_d_ values for OSGEPL1 binding to hmtRNA^Thr^ and its variants | |
| --- | --- |
| tRNA | *K*_d_ (μM)^a^ |
| hmtRNA^Thr^  U34C  U34G | 6.7 ± 1.0  5.5 ± 0.4  4.1 ± 0.3 |
| ^a^*K*_d_ values were obtained by fitting the processed data to a binding/dissociation curve with the 1:1 model in the Octet analysis software with R^2^ >0.99 for fitting. | |

| **Supplementary Table 3. *K*_d_ values for binding of OSGEPL1 and its variants to hmtRNA^Thr^** | |
| --- | --- |
| Proteins | *K*_d_ (μM)^a^ |
| OSGEPL1 | 6.7 ± 1.0 |
| K203Q | 14 ± 0.2 |
| K203E | 8.5 ± 0.3 |
| K203A | 29 ± 5.1 |
| K203R | 3.6 ± 0.1 |
| K299Q | 6.8 ± 0.3 |
| ^a^*K*_d_ values were obtained by fitting the processed data to a binding/dissociation curve with the 1:1 model in the Octet analysis software with R^2^ >0.99 for fitting. | |
